# Neuroanatomical underpinnings of autism symptomatology in carriers and non-carriers of the 22q11.2 microdeletion

**DOI:** 10.1186/s13229-020-00356-z

**Published:** 2020-06-08

**Authors:** Maria Gudbrandsen, Anke Bletsch, Caroline Mann, Eileen Daly, Clodagh M. Murphy, Vladimira Stoencheva, Charlotte E. Blackmore, Maria Rogdaki, Leila Kushan, Carrie E. Bearden, Declan G. M. Murphy, Michael C. Craig, Christine Ecker

**Affiliations:** 1 Department of Forensic and Neurodevelopmental Sciences, and the Sackler Institute for Translational Neurodevelopmental Sciences, Institute of Psychiatry, Psychology and Neuroscience, King’s College, London, UK; 2 Department of Child and Adolescent Psychiatry, Psychosomatics and Psychotherapy, University Hospital, Goethe University, Frankfurt, Germany; 3 Brain Imaging Center, Goethe University, Frankfurt, Germany; 4 Behavioural Genetics Clinic, Adult Autism and AHDH Services, Behavioural and Developmental Clinical Academic Group, South London and Maudsley Foundation, NHS, London, UK; 5 Psychiatric Imaging Group, MRC London Institute of Medical Sciences, Imperial College, London, UK; 6 Department of Psychosis Studies, Institute of Psychiatry, Psychology and Neuroscience, King’s College, London, UK; 7 Department of Child and Adolescent Psychiatry, Institute of Psychiatry, Psychology and Neuroscience, King’s College, London, UK; 8 Department of Psychiatry and Biobehavioral Sciences, Semel Institute for Neuroscience and Human Behavior and Department of Psychology, University of California-Los Angeles, Los Angeles, CA USA; 9 National Autism Unit, Bethlem Royal Hospital, London, UK

**Keywords:** 22q11.2 deletion syndrome, Autism spectrum disorder, Brain anatomy, Neurodevelopment, Surface-based anatomy

## Abstract

**Background:**

A crucial step to understanding the mechanistic underpinnings of autism spectrum disorder (ASD), is to examine if the biological underpinnings of ASD in genetic high-risk conditions, like 22q11.2 deletion syndrome (22q11.2DS), are similar to those in idiopathic illness. This study aimed to examine if ASD symptomatology in 22q11.2DS is underpinned by the same—or distinct—neural systems that mediate these symptoms in non-deletion carriers.

**Methods:**

We examined vertex-wise estimates of cortical volume (CV), surface area (SA), and cortical thickness across 131 individuals between 6 and 25 years of age including (1) 50 individuals with 22q11.2DS, out of which *n* = 25 had a diagnosis of ASD, (2) 40 non-carriers of the microdeletion with a diagnosis of ASD (i.e., idiopathic ASD), and (3) 41 typically developing (TD) controls. We employed a 2-by-2 factorial design to identify neuroanatomical variability associated with the main effects of 22q11.2DS and ASD, as well as their interaction. Further, using canonical correlation analysis (CCA), we compared neuroanatomical variability associated with the complex (i.e., multivariate) clinical phenotype of ASD between 22q11.2 deletion carriers and non-carriers.

**Results:**

The set of brain regions associated with the main effect of 22q11.2DS was distinct from the neuroanatomical underpinnings of the main effect of ASD. Moreover, significant 22q11.2DS-by-ASD interactions were observed for CV and SA in the dorsolateral prefrontal cortex, precentral gyrus, and posterior cingulate cortex, suggesting that the neuroanatomy of ASD is significantly modulated by 22q11.2DS (*p* < 0.01). We further established that the multivariate patterns of neuroanatomical variability associated with differences in symptom profiles significantly differed between 22q11.2 deletion carriers and non-carriers.

**Limitations:**

We employed a multicenter design to overcome single-site recruitment limitations; however, FreeSurfer-derived measures of surface anatomy have been shown to be highly reliable across scanner platforms and field strengths. Further, we controlled for gender to address the differing distribution between idiopathic ASD individuals and the other groups. Nonetheless, the gender distribution in our sample reflects that of the respective populations, adding to the generalizability of our results. Last, we included individuals with a relatively wide age range (i.e., 6–25 years).

**Conclusions:**

Our findings indicate that neuroanatomical correlates of ASD symptomatology in carriers of the 22q11.2 microdeletion diverge from those in idiopathic ASD.

## Background

22q11.2 deletion syndrome (22q11.2DS) is a genetic condition resulting from a microdeletion at the q11.2 band of chromosome 22 [1]. The estimated prevalence of 22q11.2DS is about 1 in 4000 [2], with an equal proportion of affected males and females [3]. This makes 22q11.2DS the most common microdeletion syndrome in the general population [4, 5]. While all individuals with 22q11.2DS display a deletion within the same locus of chromosome 22, the phenotypic consequences of the deletion are both complex and variable [6]. These encompass congenital and somatic features, as well as neuropsychiatric conditions. For example, there are high rates of comorbid neuropsychiatric conditions such as the following: autism spectrum disorder (ASD, ranging from 18 to 58% until early adulthood) [7–10], attention deficit hyperactivity disorder (ADHD, 37% in childhood) [7], anxiety disorders (approximately 35% in childhood and adolescence) [7], psychotic symptoms (57% in adolescence and young adulthood) [11, 12], and psychotic spectrum disorders (around 41% in adulthood) [7, 13, 14]. These typically occur at different developmental stages, making the clinical phenotype of 22q11.2DS highly heterogeneous not only between carriers, but also within affected individuals over time.

Some evidence suggests that the complex clinical phenotypes associated with 22q11.2DS represent distinct clinical outcomes that are underpinned by separable neurobiological mechanisms. For example, it has been shown that inter-individual variability in brain structure accompanies the occurrence and severity of positive psychotic symptoms in 22q11.2DS [15]. Yet, it remains largely unknown whether the neuropsychiatric symptoms that are commonly observed in 22q11.2DS are also mediated by the same neural mechanisms that underpin these symptoms in individuals without the microdeletion. This particularly applies to ASD symptomatology, which compared to psychotic symptomatology remains currently underexplored. Despite the high prevalence, only three neuroimaging studies to date have examined the neuroanatomical underpinnings of ASD in the brains of 22q11.2DS individuals. Two of these studies report differences in right amygdala volume between 22q11.2DS individuals with and without ASD symptomatology, but there is no consensus yet with regard to the direction of the effect (i.e., enlarged or reduced in ASD) [9, 10]. A recent study by our group also reported differences in cortical volume (CV) and surface area (SA) between 22q11.2DS individuals with and without ASD symptomatology. These differences were predominantly observed in parieto-temporal regions as well as in the posterior cingulate and dorsolateral prefrontal cortices, brain regions that have previously been linked to wider autistic symptoms and traits [16]. Preliminary evidence therefore suggests that 22q11.2DS individuals with ASD symptomatology are neuroanatomically distinct from those without ASD, and may represent a distinct neurobiological subgroup. None of these studies, however, have included a comparison to individuals with idiopathic ASD and it therefore remains unknown how closely the neurobiological phenotype of ASD in 22q11.2DS resembles the ASD phenotype in those without the microdeletion. Further, if we are to better identify how microdeletions impact on the phenotype of ASD, we need to understand if abnormalities in ASD individuals with and without 22q11.2DS are shared (or not).

This study aimed to examine the neuroanatomical underpinnings of ASD symptomatology across disorders using a categorical approach that allowed us to establish the extent to which 22q11.2DS modulates the neuroanatomy of ASD. Based on prior evidence [16], it was hypothesized that ASD symptomatology in 22q11.2DS is not simply due to a higher neuroanatomical “load” or affection status (i.e., more severe behavioral impairments associated with more pronounced neuroanatomical differences), but instead significantly interacts with the microdeletion to give rise to a distinct neuroanatomical brain phenotype. Moreover, in a second analysis step, we aimed to consolidate the results of the categorical approach, where ASD is treated as a “fixed-effect” variable based on diagnostic labels, with a multivariate dimensional approach using canonical correlation analysis (CCA), where ASD is considered a complex clinical construct or phenotypic trait spanned by multiple symptom domains across disorders. This allowed us to link the complex clinical phenotype(s) of ASD with neuroanatomical variability in multiple brain regions across disorders, and to compare their multivariate association between groups.

## Methods

### Participants

The total sample consisted of 131 individuals between 6 and 25 years of age, including (1) 50 individuals with 22q11.2DS, where *n* = 25 had a diagnosis of ASD (22q11.ASD) and *n* = 25 individuals did not (22q11.nonASD); (2) 40 non-deletion individuals with a diagnosis of ASD (i.e., idiopathic ASD); and (3) 41 typically developing (TD) controls without the microdeletion. The 22q11.2DS group was recruited in London, UK, and in Los Angeles, USA; and the idiopathic ASD group in Frankfurt, Germany. The TD controls were pooled at equal proportions across all three sites (see Table 1 and Supplementary Methods 1). The 22q11.2 microdeletion was confirmed by in situ hybridization (FISH) or microarray. All non.22q11.2DS participants were screened for somatic features associated with the microdeletion (i.e., cleft palate abnormalities, heart surgery, characteristic facial abnormalities, and hypoparathyroidism), as well as other disorders associated with 22q11.2DS. As none of these individuals displayed somatic abnormalities associated with the 22q11.2 microdeletion, we did not perform genetic testing. ASD was assessed using the Autism Diagnostic Interview-Revised (ADI-R) [17] and the Autism Diagnostic Observation Schedule (ADOS; for information on the calculation of ADOS Calibrated Severity Scores (CSS) see Supplementary Methods 2) [18, 19]. In accordance with previously published studies examining ASD symptomatology in 22q11.2DS [9, 16], all individuals with ASD met diagnostic cutoffs in the reciprocal social interaction (cutoff = 10) and communication domain (cutoff = 8) of the ADI-R, but were allowed to fall below threshold in the repetitive behaviors domain (cutoff = 3). The 22q11.2DS sample has previously been described in Gudbrandsen et al. [16]. To capture autism along a continuum consisting of multiple symptom domains, we also administered the Social Responsiveness Scale (SRS) [20, 21] in all participants, including individuals without a diagnosis of ASD. Overall intellectual ability was assessed using the Wechsler Abbreviated Scale of Intelligence (WASI) [22]. All participants or parents for those under 18 years of age gave informed written consent in accordance with ethics approval by the respective institutional ethics boards. For further details on demographics and exclusion criteria see Supplementary Methods 1.

**Table 1:** Participant demographics and global brain measures

	22q11.nonASD		22q11.ASD		ASD		Controls		Test statistic	
	(*n* = 25 [11♂, 14♀])		(*n* = 25 [13♂, 12♀])		(*n* = 40 [32♂, 8♀])		(*n* = 41 [23♂, 18♀])		*χ*^2^	*p*
IoPPN/UCLA/Frankfurt	8/17/0		17/8/0		0/0/40		14/14/13		105.03	< 0.001
									*F*	*p*
Age [years]	14 ± 6	(6–25)	15 ± 4	(7–23)	15 ± 2	(11–18)	14 ± 4	(7–24)	0.18	0.91
Full-scale IQ	86 ± 15	(60–116)	81 ± 12	(61–112)	96 ± 13	(64–116)	104 ± 11	(76–123)	21.01	< 0.001
ADI-R *social*^1^	5 ± 3	(1–9)	19 ± 5	(9–28)	17 ± 4	(9–27)	n/a	n/a	95.93	< 0.001
ADI-R *communication*^1^	6 ± 4	(0–16)	14 ± 4	(8–24)	13 ± 4	(5–23)	n/a	n/a	29.98	< 0.00
ADI-R *repetitive*^1^	1 ± 2	(0–8)	3 ± 3	(0–10)	5 ± 2	(1–10)	n/a	n/a	16.8	< 0.001
ADOS *CSS*^1^	3 ± 2	(1–8)	6 ± 3	(1–10)	6 ± 3	(1–10)	n/a	n/a	9.3	< 0.001
SRS *total score*	55 ± 26	(16–103)	101 ± 33	(41–174)	95 ± 29	(42–159)	23 ± 19	(0–75)	69.38	< 0.001
SRS *repetitive*	9 ± 6	(1–22)	17 ± 7	(7–32)	16 ± 7	(0–34)	2 ± 3	(0–10)	49.31	< 0.001
Total cortical volume [L]	0.66 ± 0.08	(0.41–0.89)	0.68 ± 0.07	(0.52–0.78)	0.75 ± 0.07	(0.60–0.92)	0.73 ± 0.07	(0.59–0.90)	10.56	< 0.001
Total surface area [m^2^]	0.20 ± 0.02	(0.13–0.25)	0.21 ± 0.02	(0.17–0.24)	0.23 ± 0.02	(0.18–0.27)	0.22 ± 0.02	(0.19–0.27)	12.41	< 0.001
Mean cortical thickness [mm]	2.78 ± 0.13	(2.59–3.06)	2.77 ± 0.10	(2.60–2.96)	2.71 ± 0.10	(2.51–2.93)	2.71 ± 0.10	(2.48–3.01)	3.74	< 0.05

Data expressed as mean ± standard deviation (range); (1) data based on 89 individuals

*ADI-R* Autism Diagnostic Interview-Revised, *ADOS* Autism Diagnostic Observation Schedule, *CCS* Calibrated Severity Score, *SRS* Social Responsiveness Scale

### MRI data acquisition

For image acquisition, we employed a contemporary MRI scanner operating at 3 T (Siemens Trio in Frankfurt and Los Angeles (UCLA), and a Signa GE Medical System in London (IoPPN)). High-resolution structural ADNI MPRAGE sequences were acquired with full head coverage. At the IoPPN, 166 contiguous slices (1.2 mm thickness, with 1.2 × 1.2 mm in-plane resolution) were acquired using a repetition time/echo time (TR/TE) of 7/2.9 ms (flip angle = 8°, FOV = 26 cm). At UCLA, 160 contiguous slices (1.2 mm thickness, with 1.2 × 1.2 mm in-plane resolution) were acquired using a TR/TE of 2300/2.9 ms (flip angle = 8°, FOV = 26 cm). In Frankfurt, 176 contiguous slices (1.0 mm thickness, with 1.0 × 1.0 mm in-plane resolution) were acquired using a TR/TE of 2300/2.2 ms (flip angle = 9°, FOV = 26 cm). Consistent image quality was ensured by a semi-automated quality control procedure at all sites, including a stringent pre-processing pipeline.

### Cortical surface reconstruction using FreeSurfer

FreeSurfer v6.0.0 software (http://surfer.nmr.mgh.harvard.edu/) was used to derive models of the cortical surface for each T_1_-weighted image. These well validated and fully automated procedures have been extensively described elsewhere [23–27]. Measures of cortical thickness (CT) were computed as the closest distance from the gray-white matter boundary to the gray matter-cerebrospinal fluid boundary at each vertex on the tessellated surface [25]. Vertex-based estimates of SA were derived as outlined by Winkler et al. [28]. For each participant, we also computed mean CT across the entire brain, as well as total brain volume and total SA. To improve the ability to detect population changes, each parameter was smoothed using a 5-mm surface-based smoothing kernel. Details on excluded scans, quality assessments, and manual editing of surface models are described in the Supplementary Methods 3.

### Statistical analyses

Statistical analyses were conducted using the SurfStat toolbox (http: //www.math.mcgill.ca/keith/surfstat/) for Matlab (R2017b; MathWorks). Between-group differences in age, full-scale IQ, ASD symptom severity, and total brain measures were assessed via analyses of variance (ANOVA) with group as categorical fixed-effect factor. Pair-wise differences between subgroups were examined post hoc using Scheffé test to correct for multiple comparisons in R (R3.5.2; see Supplementary Tables S1). Parameter estimates for vertex-based measures of CV, SA, and CT were estimated by regression of a general linear model (GLM) for each vertex *i*, with (1) group membership (i.e., having the microdeletion and/or having a diagnosis of ASD), gender, and site as categorical fixed-effect factors; (2) a 22q11.2DS-by-ASD interaction term; and (3) full-scale IQ, a linear and a quadratic age term, and the respective total brain measure (total brain volume for CV, total SA for SA, and mean CT for CT) as continuous covariates, so that

$$ {Y}_i={\upbeta}_0+{\upbeta}_122\mathrm{q}11.2\mathrm{DS}+{\upbeta}_2\mathrm{ASD}+{\upbeta}_3\left(22q11.2 DSxASD\right)+{\upbeta}_4\mathrm{Gender}+{\upbeta}_5\mathrm{Site}+{\upbeta}_6\mathrm{IQ}+{\upbeta}_7\mathrm{Age}+{\upbeta}_8\mathrm{Ag}{e}^2+{\upbeta}_9\mathrm{Total}\kern0.17em \mathrm{Brain}+{\upvarepsilon}_i, $$

where *ε*_*i*_ is the residual error at vertex *i*. All included continuous covariates were mean centered across groups to improve interpretability of the coefficients. We examined between-group differences for the main effect of 22q11.2DS, estimated from the corresponding coefficient β_1_, as well as the main effect of ASD, estimated from the corresponding coefficient β_2_, normalized by the corresponding standard error respectively. We further examined the interaction effect between 22q11.2DS and ASD (coefficient β_3_) across parameters. Due to reasons of completeness, we also examined the main effects of ASD and 22q11.2DS in separate samples, comparing them to TD controls only (see Supplementary Figs. S1 & S2).

Corrections for multiple comparisons across the whole brain were performed using “random field theory” (RFT)-based cluster analysis for non-isotropic images using a cluster-based significance threshold of *p* < 0.05 (2-tailed) [29]. An RFT-based cluster correction was chosen over a permutation-based approach given its computational efficiency particularly for complex GLMs as utilized in the present study, and to make the results comparable to previous findings by our group [16, 30]. Uncorrected *t* maps and effect size images for the main effects are presented within the Supplementary Material (see Supplementary Figs. S3 & S4). As the 22q11.ASD and idiopathic ASD groups differed in symptom severity in the repetitive behavior domain of the ADI-R, we also performed the analysis covarying for the SRS Restricted Interests and Repetitive Behavior subscale. Further, given the large phenotypic heterogeneity typically associated with idiopathic ASD, we also tested for homogeneity of variances using the Levene’s test comparing the 22q11.ASD group to the idiopathic ASD groups (see Supplementary Fig. S5). Last, we reran the analysis with stricter matching for age and gender distribution (see Supplementary Table 2, and Supplementary Fig. S6).

### Canonical correlation analysis (CCA)

In a second analysis step, we examined differences in the neural systems mediating autistic symptoms in 22q11.2DS individuals and individuals without the microdeletion (abbreviated as non22q11.2DS) within the dimensional framework of CCA. Here, ASD was not treated as a categorical fixed effect across groups, but as multivariate latent trait construct that is spanned by inter-individual differences in symptom profile. The general framework of CCA is well described elsewhere ([31]; see also Supplementary Methods 4). In the present study, we examined the relationship between neuroanatomical variability in CV, SA, and CT as predictors (*X*_*n* × *p*_), and the five SRS subdomain scores in social awareness (SAW), social cognition (SCG), social communication (SCM), social motivation (SM), and restricted and repetitive behaviors (RRB) as clinical outcomes (*Y*_*n* × *q*_, where *q* = 5) (see Supplementary Fig. S7 for schematic illustration). To reduce the large number of vertex-based neuroanatomical features to a smaller subset of regions, we initially parcellated the cortex into a set of 34 cortical regions per hemisphere using the Desikan-Killiany cortical parcellation atlas [32], after correcting the data for linear and quadratic age effects, gender, site, full-scale IQ, and total brain measures across groups, which resulted in a set of *n* = 204 neuroanatomical features in total. As classical CCA assumes that the number of features is less than the number of samples (i.e., *n* ≤ max(*p*, *q*)), we employed different feature selection approaches to identify a set of neuroanatomical features that are most relevant to the prediction of the clinical SRS subdomain scores. The different feature selection approaches as well as the resulting subsets of neuroanatomical features are described in detail in the Supplementary Materials (see Supplementary Methods Section 5). We then based the analysis presented within the main manuscript on the feature selection approach that provided the largest subset of clinically relevant neuroanatomical features. This was a stepwise regression procedure with Akaike information criterion (AIC)-based model selection [33], which highlighted a set of *p* = 63 neuroanatomical features in total (see Supplementary Fig. S8).

CCA was initially employed as a *backward model* that derives canonical variates as functions of the observed data based on the matrix of coefficients or weights (i.e., $\hat{x}={\boldsymbol{W}}^Tx$). To estimate coefficients, we applied CCA to all individuals in our sample (i.e., *n* = 131), resulting in a set of *i* = 5 canonical variate pairs, which represent the relationship between neuroanatomical variability and inter-individual differences in autism symptom profiles. The RV-coefficient [34] was firstly examined to test for the overall statistical significance of the multivariate association between *X* and *Y*. Moreover, the significance of the full canonical model was evaluated using Wilks’ lambda ( [35]) and Pillai’s Trace [36]. To identify the number of significant canonical variate pairs for the subsequent comparison between groups, a dimension reduction analysis was performed. Here, we explored the percentage of variance explained by each neuroanatomical and clinical canonical variate using Rao’s *F* test [37] and Bartlett’s chi-squared test [38]. To enable a better interpretation of the data, canonical variates were sorted in descending order based on their level of statistical significance, and the fraction of total clinical variance explained by each canonical variate pair (i.e., canonical variate adequacies for clinical measures). The results of the CCA were visualized based on the canonical variate scores ($\hat{X},\hat{Y}$), and the matrix of structural correlations or “loadings” (**Λ** with element *λ*), which represent the association between the observed neuroanatomical and clinical data with their respective canonical variates ($\mathrm{i}.\mathrm{e}.,{\lambda}_{p,i}=\mathrm{cor}\left({x}_p,{\hat{x}}_i\right)$), where *p* denotes the canonical variate number.

To examine differences in the spatially distributed patterns of neuroanatomical variability underpinning autism symptom profiles in carriers and non-carriers of the 22q11.2 microdeletion, we utilized the model coefficients and canonical variate scores resulting from the *backward model*, to model the association between the observed data and the derived canonical variates within groups by applying a *forward model* of the general form $x=\boldsymbol{\Lambda} \hat{x}+\varepsilon$, where *ε* denotes the residual error [39]. If estimated latent factors are uncorrelated, which is the case in CCA, it has been shown that

$$ \boldsymbol{\Lambda} \propto {X}^TX\ \mathbf{W}={X}^T\hat{X}=\operatorname{cov}\left(X,\hat{X}\right) $$

which equals the correlation (i.e., loading) between *X* and $\hat{X}$ if variables are standardized [40]. The *forward model* was applied twice, after splitting the data into carriers and non-carriers of the 22q11.2 microdeletion, which resulted in a new set of group-specific clinical and neuroanatomical loadings. The *Tucker*’*s congruence coefficient* [41] was used to compare the loading matrices between groups, where a congruence coefficient in the range of [0.85–0.94] corresponds to a *fair* similarity, and a value > 0.95 indicates that the structure of two factors almost equal [42]. We also tested individual loading pairs for a statistical between-group difference using *Fisher-Z* transformation for independent correlation coefficients (*p* < 0.05, one-tailed) [43]. Last, we examined the reliability of our findings across different feature selection approaches (see Supplementary Methods 5 for further details). All statistical analyses were performed using RStudio Version 1.2 (https://www.rstudio.com/products/rstudio/) using the CCA results provided by yacca: Yet Another Canonical Correlation Analysis Package toolbox (https://CRAN.R-project.org/package=yacca).

### Data availability

Further details on the data and utilized software are available upon request from the corresponding author. The full set of raw data is not currently publicly available due to ethical restrictions. However, a subset of the sample can be made available upon request.

## Results

### Participant demographics, diagnostic group, and global brain measures

There were no significant between-group differences in participants’ age. However, groups differed significantly in gender distribution (*χ*^2^(3) = 2.26, *p* = 0.016), with a lower percentage of females in the idiopathic ASD group relative to the other subgroups, and in full-scale IQ (*F*(3) = 21.01, *p* < 0.001), with TD controls scoring higher than all other groups, and individuals with 22q11.2DS having a lower IQ than individuals with idiopathic ASD. Further, we found a significant effect of group for total brain volume (*F*(3) = 10.56, *p* < 0.001) and total SA (*F*(3) = 12.41, *p* < 0.001), with both 22q11.2DS groups having a significantly lower total volume and area compared to both idiopathic ASD and TD controls (*p* < 0.05 for all pair-wise comparisons). Last, there was a significant effect of group for mean CT (*F*(3) = 3.74, *p* < 0.05) across the cortex, with idiopathic ASD individuals having a trend towards reduced CT compared to 22q11.nonASD individuals (*p* = 0.088), while no other pair-wise comparison was significant (see Table 1 and Supplementary Tables S1 for all pair-wise comparisons). We thus co-varied for gender, full-scale IQ, and respective total brain measure in all subsequent analyses.

### Results of the categorical fixed-effects analyses

#### Main effect of 22q11.2DS on CV, SA, and CT

**Fig. 1:**
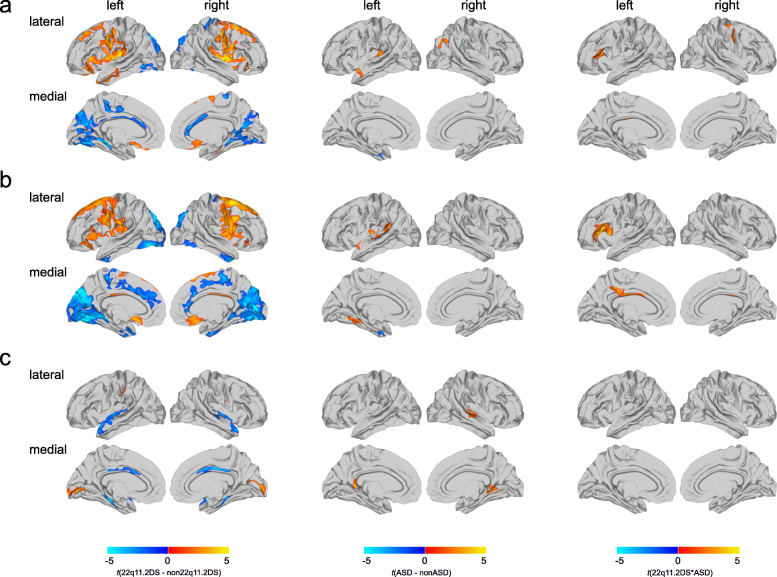
Categorical results for main effect of 22q11.2DS, main effect of ASD, and 22q11.2DS-by-ASD interaction effect*.***a** Significant differences in cortical volume (CV), **b** surface area (SA), and **c** cortical thickness (CT) for the main effect of 22q11.2DS (left panel), the main effect of ASD (middle panel), and for the 22q11.2DS-by-ASD interaction (right panel). Displayed are the random field theory (RFT)-based cluster corrected (*p* < 0.05, 2-tailed) difference maps following multiple comparisons, where increased parameter estimates in 22q11.2DS (or ASD) are marked in red to yellow, and decreased parameters are marked in blue to cyan

Significant neuroanatomical differences between 22q11.2 deletion carriers (i.e., 22q11.2DS with and without ASD) and non-carriers (i.e., idiopathic ASD and TD controls) were observed in several large clusters distributed across the cortex. More specifically, CV was increased in 22q11.2DS in the bilateral superior frontal cortex, the lateral and medial orbitofrontal cortex, the pre- and postcentral gyrus, the insula, and the supramarginal gyrus, with increases being driven by a commensurate increase in SA. Increased CV in 22q11.2DS was also observed in the left middle temporal gyrus, while increased SA was further observed in the left superior temporal gyrus and the left posterior cingulate cortex (PCC). In contrast, CV was decreased in 22q11.2DS in a large cluster centered on the bilateral medial occipital and temporal lobes, as well as in the bilateral anterior cingulate cortex, and the pre- and postcentral gyrus, accompanied by commensurate decreases in SA. Further decreases in SA were observed in the bilateral dorsal anterior cingulate area and inferior temporal gyri. Last, we identified increased CT in 22q11.2DS in some scattered regions, including the bilateral lateral occipital cortex, the right postcentral gyrus, and the left supramarginal gyrus, whereas decreases in CT were observed in the bilateral superior temporal lobes, the parahippocampal gyri, and the posterior cingulate cortex (see Fig. 1, Supplementary Fig. S3, and Supplementary Table S3). Effect size images for the main effect of 22q11.2DS are shown in Supplementary Fig. S4. A similar pattern of effects was also obtained when comparing the 22q11.2DS individuals to TD controls only (see Supplementary Fig. S1), and when strictly matching for age and gender (see Supplementary Fig. S6).

#### Main effect of ASD on CV, SA, and CT

For the main effect of ASD, we established that individuals with ASD symptomatology (i.e., individuals with idiopathic ASD and 22q11.ASD) were neuroanatomically distinct from those without (i.e., compared to TD controls and 22q11.nonASD), with significantly increased CV in the left insula and left superior temporal gyrus, accompanied by a more widespread increase in SA, also spanning the fusiform, parahippocampal, lingual, and supramarginal gyri. CV was further increased in the right inferior parietal cortex in ASD. In contrast, decreases in CV in ASD were observed in the left entorhinal cortex, accompanied by a commensurate decrease in SA that was more pronounced and also implicated the left fusiform gyrus. For measures of CT, individuals with ASD showed significant increases in the right isthmus cingulate cortex and the right superior temporal gyrus (see Fig. 1, Supplementary Fig. S3, and Supplementary Table S4). Effect size images for the main effect of ASD are shown in Supplementary Fig. S4. A similar pattern of effects was also obtained when comparing the idiopathic ASD individuals to TD controls only (see Supplementary Fig. S2), and when strictly matching for age and gender (see Supplementary Fig. S6).

#### Significant interactions between 22q11.2DS and ASD

In addition to the main effects, we observed significant interactions between 22q11.2DS and ASD for measures of CV and SA. These were located in the left dorsolateral prefrontal cortex (DLPFC) for both, CV and SA, as well as in the right precentral gyrus for CV only, and in the left PCC for SA only (see Fig. 1, Supplementary Fig. S3, and Supplementary Table S5). Effect size images for the 22q11.2DS-by-ASD interaction are shown in Supplementary Fig. S4. In significant clusters, ASD was associated with increased CV and/or SA in 22q11.2DS (i.e., 22q11.ASD > 22q11.nonASD), but reduced CV and/or SA in individuals without the microdeletion (i.e., idiopathic ASD < TD controls). In the DLPFC and PCC, individuals with 22q11.nonASD were the most affected on the neuroanatomical level (i.e., had the most reduced CV and/or SA relative to all other groups), while both ASD groups were comparable in terms of their mean CV and/or SA (22q11.nonASD < 22q11.ASD = ASD ≤ TD controls). In the precentral cluster exclusively, 22q11.ASD individuals had the largest mean CV compared to all other groups, with the mean of 22q11.nonASD individuals being between idiopathic ASD and TD controls (for boxplots see Supplementary Fig. S9). As the 22q11.ASD and idiopathic ASD groups differed in symptom severity in the repetitive behavior domain of the ADI-R, we also performed the analysis covarying for the SRS Restricted Interests and Repetitive Behavior subscale. However, the patterns of significant 22q11.2DS-by-ASD interactions remained unchanged overall (see Supplementary Fig. S10). In regions with significant 22q11.2DS-by-ASD interactions, there were also no significant differences in variance between the idiopathic ASD individuals and the 22q11.ASD group (see Supplementary Fig. S5), and very little effect of age and gender (see Supplementary Fig. S6).

### Results of the CCA

**Fig. 2:**
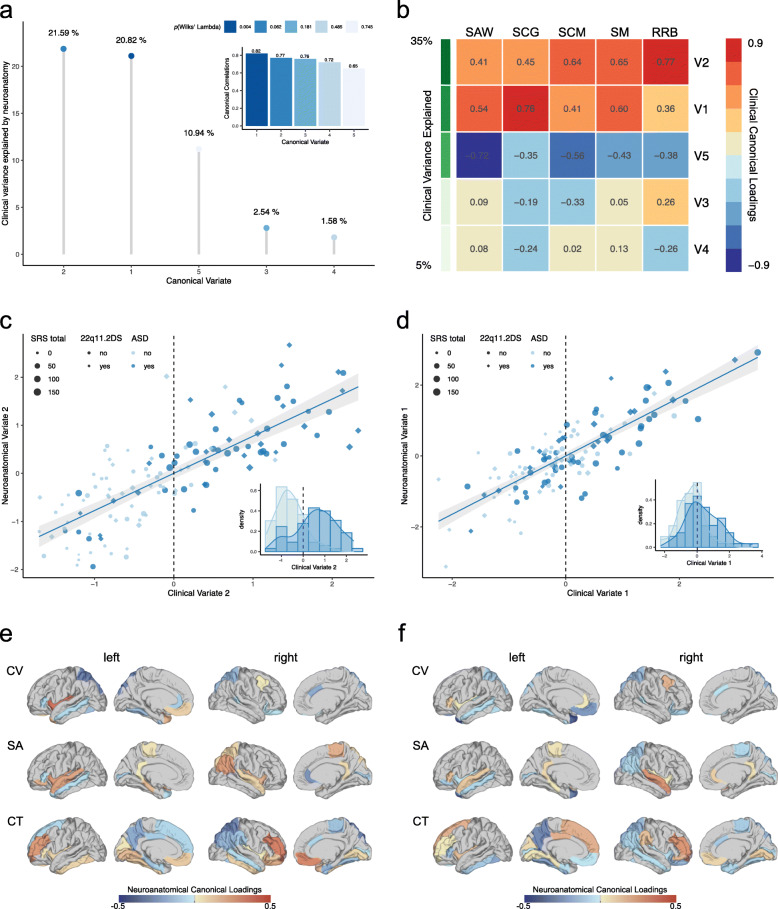
Results of the CCA across all individuals within our sample*.***a** Canonical correlations (subplot) and canonical variates sorted in descending order based on their canonical correlations, and based on the percentage of clinical variance explained; **b** clinical canonical loadings depicting correlations between each of the five clinical canonical variates (V1-V5) and the five SRS subdomain scores in social awareness (SAW), social cognition (SCG), social communication (SCM), social motivation (SM), and restricted and repetitive behaviors (RRB). Canonical variates are sorted in descending order based on the percentage of explained clinical variance, as indicated in shades of green; **c** and **d** scatter plots depicting individual observations based on their scores on the second (**c**) and first (**d**) canonical variate, which explained the largest percentage of clinical variance. Data points are colored (ASD: yes vs. no) and shaped (22q11.2DS: yes vs. no) by group membership and sized by the individual’s total SRS score; **e** and **f** display canonical loadings of each neuroanatomical feature (i.e., cortical volume (CV), surface area (SA), and cortical thickness (CT)) on the second (**e**) and first (**f**) canonical variate

Initially, CCA was performed across all individuals within our sample (i.e., carriers and non-carriers of the 22q11.2 microdeletion). Here, we observed a significant multivariate association between the 63 regional measures of brain anatomy highlighted to be of importance by the stepwise variable selection approach, and the five symptom domains of the SRS (RV_coef_ = 0.082, *p* < 0.001; see Supplementary Fig. S11 for distribution of SRS total and subdomain scores across groups). Based on the number of clinical predictors (*q* = 5), the CCA yielded five canonical variate pairs with the canonical correlations of 0.822, 0.772, 0.764, 0.724, and 0.653 for each successive canonical variate pair, respectively (see Fig. 2a). Collectively, the full model including all canonical variates was statistically significant using Wilks’ *λ* = 0.015 (*F*(315,319) = 1.35, *p* < 0.01) and Pillai’s trace = 2.81 (*F*(315,335) = 1.36, *p* < 0.01). As Wilks’ *λ* indicates the variance unaccounted for by the model, the R-square type (*ρ*^2^) effect size of the model was 0.985 (i.e., 1-λ), which means that the full model explained about 98.5% of the variance shared between measures of neuroanatomy and clinical symptom profile. Moreover, the total variance in SRS scores that could be explained by neuroanatomical variation was 57.47%, which only the first two neuroanatomical canonical variates contributed to significantly (20.82% and 21.59%, respectively; see Fig. 2a). Out of all canonical variates, the 1st (Bartlett’s *χ*^2^(315) = 402.24, *p* < 0.001) and the 2nd (Bartlett’s *χ*^2^(248) = 294.58, *p* < 0.05) were also statistically significant, with the 1st clinical canonical variate explaining a total of 30.80%, and the 2nd clinical canonical variate explaining a total of 36.19% of variability within the set of clinical variables on their own (clinical canonical variate adequacy, see Fig. 2b). Thus, given the *ρ*^2^ effects for each canonical variate pair, only the first two pairs were considered noteworthy in the context of the present study. Both clinical canonical variates, and the 2nd canonical variate in particular, also provided a good discrimination between individuals with and without ASD (see Fig. 2c, d). Figure 2 e and f show the canonical loadings (*λ*_*C*_) for each neuroanatomical predictor on the cortical surface, which highlights the set of brain regions maximally correlated with the 2nd (e) and 1st (f) neuroanatomical canonical variate. As expected, high positive loadings (i.e., > 0.25) were observed in many regions of the social brain including the right medial orbitofrontal lobe (CT, *λ*_*C2*_ = 0.28), the right rostral middle frontal gyrus (CT, *λ*_*C2*_ = 0.30), the left insula (CV, *λ*_*C2*_ = 0.34), and the left transverse temporal lobe (CV, *λ*_*C2*_ = 0.28). High negative loadings were observed in the left precuneus (CT, *λ*_*C1*_ = − 0.27), the bilateral superior parietal lobes (CT, right: *λ*_*C2*_ = − 0.28; CV, left: *λ*_*C2*_ = − 0.28), and the left temporal pole (SA, *λ*_*C1*_ = − 0.30; CV, *λ*_*C1*_ = − 0.38).

**Fig. 3:**
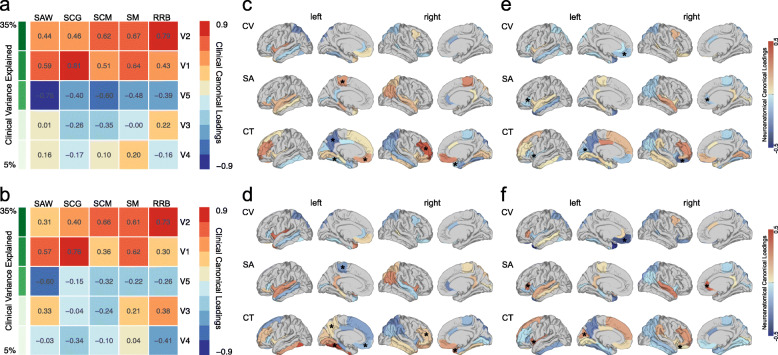
Comparison of factor loadings between carriers and non-carriers of the 22q11.2 microdeletion. Figures (**a**) and (**b**) display clinical canonical loadings plot depicting correlations between each of the five clinical canonical variates and each of the five SRS subdomain scores in social awareness (SAW), social cognition (SCG), social communication (SCM), social motivation (SM), and restricted and repetitive behaviors (RRB) within **a** all non22q11.2DS individuals (i.e., idiopathic ASD and TD controls) and within **b** all 22q11.2DS individuals (i.e., 22q11.ASD and 22q11.nonASD). Canonical variates are sorted in descending order based on the percentage of explained clinical variance as indicated in shades of green; **b** and **c** canonical loadings of each neuroanatomical predictor (i.e., cortical volume (CV), surface area (SA), and cortical thickness (CT)) on canonical variate 2 within **c** all non22q11.2DS individuals and **d** all 22q11.2DS individuals; **e** and **f** canonical loadings of each neuroanatomical predictor (i.e., CV, SA, and CT) on canonical variate 1 within **e** all non22q11.2DS individuals and **f** all 22q11.2DS individuals. Brain regions with a significant between-group difference in brain loadings between non22q11.2DS and 22q11.2DS individuals are indicated with an asterisk

After fitting the CCA in the total sample, we utilized the resulting canonical variate scores to derive group-specific factor loadings (clinical and neuroanatomical) for carriers and non-carriers of the 22q11.2 microdeletion, which were subsequently compared between groups. Overall, there was a high degree of similarity in the clinical canonical variate structure observed carriers and non-carriers, with *Tucker*’*s congruence coefficients* for the 1st and 2nd clinical covariates exceeding a value of 0.99 (see Fig. 3a, b). However, when examining the neuroanatomical underpinnings of these clinical variates between groups, we found that there was a low degree of neuroanatomical similarity overall (mean *Tucker*’*s congruence coefficient* across canonical variates = 0.336), and low levels of congruence for canonical variate 1 (*Tucker*’*s congruence coefficient* = 0.393) and variate 2 (*Tucker*’*s congruence coefficient* = 0.404). We also observed significant between-group differences in individual neuroanatomical loading pairs, which are displayed in Fig. 3c–f. More specifically, for canonical variate 2, which is the variate that explained the largest percentage of clinical variability (see Fig. 3c, d), we observed a significant difference in the loadings of the right rostral middle frontal cortex (CT; *Fisher*’*s Z* = 1.68, *p* < 0.05), the left precuneus (CT; *Fisher*’*s Z* = 2.01, *p* < 0.05), the left paracentral gyrus (SA; *Fisher*’*s Z* = 2.48, *p* < 0.01), the left medial orbitofrontal cortex (CT; *Fisher*’*s Z* = 1.90, *p* < 0.05), the left fusiform gyrus (CT; *Fisher*’*s Z* = 2.80, *p* < 0.01), and the right temporal pole (CT; *Fisher*’*s Z* = 1.78, *p* < 0.05). For canonical variate 1, the variate to explain the second most variability (Fig. 3e, f), individuals with 22q11.2DS had significantly higher neuroanatomical loadings in the left insula (CT; *Fisher*’*s Z* = 1.99, *p* < 0.05), the left cuneus (CT; *Fisher*’*s Z* = 1.95, *p* < 0.05), the right lateral orbitofrontal cortex (CT; *Fisher*’*s Z* = 1.76, *p* < 0.05), the left pars triangularis (SA; *Fisher*’*s Z* = 1.94, *p* < 0.05), and in the right rostral anterior cingulate cortex (SA; *Fisher*’*s Z* = 2.84, *p* < 0.01). Individuals with 22q11.2DS further had a more negative loading between the 1st canonical variate and the volume of the medial orbitofrontal cortex compared to non22q11.2DS individuals (CV; *Fisher*’*s Z* = 1.74, *p* < 0.05). Thus, despite the high degree of similarity in the clinical composition of autism symptoms across groups, we observed that inter-individual differences in clinical symptom profiles were underpinned by different neuroanatomical substrates in carriers and non-carriers of the 22q11.2 microdeletion.

## Discussion

This study aimed to determine whether ASD symptomatology in individuals with 22q11.2DS is underpinned by similar neuroanatomical substrates that mediate ASD symptoms in non22q11.2DS. We utilized both a categorical and a dimensional approach to (1) establish the extent to which the neuroanatomy of 22q11.DS is modulated by (i.e., significantly interacts with) having a diagnosis of ASD and to (2) compare the patterns of neuroanatomical variability that mediate the complex (i.e., multi-dimensional) clinical phenotype of ASD across disorders. Within the categorical framework, we initially established that it is possible to separate the effect of 22q11.2DS from the effect of ASD on the level of neuroanatomy as characterized by regional variability in CV, SA, and CT. Notably, we also observed significant 22q11.2DS-by-ASD interactions suggesting that, 22q11.2DS individuals who also have ASD, may represent a subgroup that is neuroanatomically distinct from 22q11.nonASD individuals, and from individuals without the microdeletion. These results were confirmed by the dimensional approach, which further highlighted that, while the complex clinical phenotype of ASD might be reduced to the same underlying (i.e., latent trait) construct across disorders, the neuroanatomical substrates associated with variation in SRS scores were different between carriers and non-carriers of the 22q11.2 microdeletion.

Neuroanatomical differences associated with the 22q11.2 microdeletion are well documented in the literature and include spatially distributed differences in CV, SA, and CT in parieto-temporal and cingulate regions, as well as the bilateral insula, parahippocampal gyrus, and DLPFC [15, 16, 44]. Here, we examined the neuroanatomy of 22q11.2DS initially within a 2 × 2 factorial design that included (1) 22q11.2DS individuals with and without ASD, (2) individuals with idiopathic ASD, and (3) TD controls. While it remains a topic of debate whether ASD should be considered a categorical “fixed-effect” variable, this design allowed us to identify a set of brain regions where neuroanatomical variability in CV, SA, and CT were uniquely attributable to either the microdeletion or having ASD. By examining the main effects of groups, we were able to demonstrate that it is possible to separate the effect of 22q11.2DS from the main effect of ASD on the neuroanatomical level based on patterns of neuroanatomical differences that included extensive and spatially distributed neuroanatomical differences across all four lobes of the cortex for the effect of 22q11.2DS, and more localized atypicalities in predominantly temporal regions for the main effect of ASD. More specifically, having ASD was linked to neuroanatomical abnormalities in the superior temporal gyrus (STG), DLPFC, insula, fusiform gyrus, parahippocampal gyrus, isthmus cingulate cortex, and entorhinal cortex. Many of these brain regions have previously been reported to be integral parts of the neural systems that mediate autistic symptoms and traits. For example, the STG has been implicated in both language and social cognition in ASD [45–47]. Further, the parahippocampal gyrus, isthmus cingulate cortex, and entorhinal cortex, are all part of the limbic system, which has been associated with impaired socioemotional and face processing in ASD [48–51]. In addition, the DLPFC is primarily associated with executive functioning, an aspect of impairment in ASD [52], and the insula has been linked to abnormalities of emotional/affective sensory functions in ASD [53]. We also examined the effect of ASD in a subsample of non-carriers of the microdeletion, i.e., in idiopathic ASD individuals relative to TD controls, with similar results. Overall, there is a strong spatial correspondence between the set of brain regions we identified as being neuroanatomically different in individuals with ASD symptomatology, including those with 22q11.2DS, and the set of brain regions mediating autistic symptoms in idiopathic ASD.

The results reported in the present study also extend the findings of a previous neuroimaging study by our group, which was conducted in a subset of this sample, where we compared 22q11.2DS individuals with ASD symptomatology to 22q11.2DS without ASD, and to TD controls [16]. Notably, the main effect of 22q11.2DS was associated predominantly with significant reductions in SA, which have been shown to contribute more significantly to commensurate differences in CV than measures of CT [54], and is also in line with previous findings in 22q11.2DS [15, 44, 55]. However, as our comparison group for the main effect of 22q11.2DS in the present study consisted of both TD controls and individuals with idiopathic ASD, the sum of squares associated with each model term was partitioned differently across studies with regard to their main effects allocation (22q11.2DS while covarying for ASD and vice versa). We therefore also examined the main effect of 22q11.2DS compared to TD controls only, which corresponded by large with our previous findings in this sample [16]. Our findings within the 22q11.2DS sample are also in agreement with a recent large scale study conducted by the 22q11.2DS ENIGMA consortium, where neuroanatomical differences in a similar set of brain regions were reported [55]. Thus, there is strong evidence to suggest that 22q11.2DS is associated with significant structural brain abnormalities, which in turn may impact on the various clinical phenotypes associated with the syndrome. Our findings indicate that 22q11.2DS and ASD have separable neuroanatomical underpinnings, but further, it suggests that, given the increased prevalence of ASD in 22q11.2DS relative to the normative population, the effects of 22q11.2DS on brain development might impact on the risk of ASD, but in itself are not sufficient to cause the condition.

Within the factorial design, it was also possible to determine to what extent the neuroanatomy of ASD is significantly modulated by 22q11.2DS (i.e., differs from individuals with 22q11.2DS without ASD and from individuals with idiopathic ASD). For example, we observed significant 22q11.2DS-by-ASD interactions in the left DLPFC and the left PCC. In these brain regions, individuals with 22q11.2DS without ASD symptomatology were the most affected, followed by 22q11.2DS individuals with ASD, individuals with idiopathic ASD, and TD controls in terms of affection status. While these interactions are complex and difficult to interpret, it seems that ASD in 22q11.2DS is not simply due to an exacerbation of the 22q11.2DS brain phenotype per se, i.e., more severe behavioral impairments associated with more pronounced neuroanatomical atypicalities. Instead, ASD symptoms in 22q11.2DS seem to be associated with a pattern of neuroanatomical differences that cannot be explained by either the microdeletion or a diagnosis of ASD alone. We also excluded the possibility that these significant interactions are driven by differences in variance between both ASD groups, as individuals with idiopathic ASD are known to be largely heterogeneous both in terms of etiology as well as clinical phenotype in comparison to the etiologically more homogeneous phenotype associated with 22q11.2DS. This implies that individuals with 22q11.2DS and ASD may constitute a distinct neuroanatomical subgroup that is neuroanatomically different from 22q11.2DS individuals without ASD, and from individuals with idiopathic ASD. Thus, although 22q11.2DS individuals with ASD share the same clinical phenotype as idiopathic ASD individuals, the neuroanatomical underpinnings appear to differ between groups, and 22q11.2DS in itself may not be sufficient to cause ASD.

While the factorial design allowed us to disentangle the effect of ASD from the main effect of 22q11.2DS, and to explore their interaction, there has been some debate whether ASD should be considered a uniform clinical construct that is common (i.e., invariant) across idiopathic and “syndromic” forms of ASD, and can hence be encoded as a categorical main effect across groups. For example, while all individuals with ASD met cutoffs in the social and communication domains of the ADI-R, not all individuals with 22q11.2DS met cutoffs in the repetitive domain (see [16] for discussion). Although our results remain stable when covarying for repetitive symptoms, it remains unclear whether the distinct neuroanatomical phenotype of 22q11.2DS with ASD is the cause or the consequence of a potentially unique clinical profile (see [56, 57]). In a second analysis step, we also employed a dimensional approach using CCA, which allowed us to treat ASD as a continuous clinical construct spanned by multiple symptom domains rather than the binary presence or absence of a diagnosis of ASD, and to examine the multivariate association between inter-individual clinical profiles and neuroanatomical variability between 22q11.2 deletion carriers and non-carriers. Here, we based our characterization of the clinical ASD phenotype on the SRS subscales, which are particularly suited to assess autistic symptoms along a continuum, with individuals with idiopathic ASD and 22q11.2DS individuals with ASD showing a very similar profile across subscales. Our findings imply that while it is possible to reduce the complex clinical ASD phenotype to two dominant latent-trait factors with comparable clinical factor structures in both carriers and non-carriers, the underlying set of brain regions that explained maximal clinical variance differed between groups. Thus, both approaches converge in suggesting that ASD symptomatology may be mediated by different neuroanatomical substrates in individuals with and without the 22q11.2 microdeletion, even when taking inter-individual variability in clinical ASD phenotypes into account.

## Limitations

Our results should be interpreted in the light of several methodological limitations of which the small sample size is the most pressing one. Although we employed a multicenter design to overcome single-site recruitment limitations, our sample size of ~ 40 individuals per group is relatively small compared to other studies, which limits the generalizability of the results and the strength of the conclusions. Even though the effects for both of our between-group comparisons are comparable to previous reports in larger samples, both in terms of size and spatial distribution, larger samples are required to replicate our findings in the future, and to provide a more robust characterization of the clinical and neuroanatomical phenotype of ASD across disorders. Moreover, the generalizability of our findings is limited by the multi-side nature of our study, which resulted in our groups being recruited at three different sites. FreeSurfer derived measures of surface anatomy have, however, been shown to be highly reliable across scanner platforms and field strengths, when MRI instrument and data processing factors are controlled for [58]. In our study, all surface reconstructions were also subjected to the same stringent type of quality assessment and pre-processing pipeline, and inter-site effects were accounted for in the statistical model. Moreover, due to the parametric nature, the dimensional approach is less biased by effects of categorical variables such as site and gender. We did also not directly test our idiopathic ASD group for copy number variations (CNVs), such as 22q11.2DS. However, we did perform extensive medical screening for somatic features associated with the 22q11.2 microdeletion across all groups (i.e., heart, palatal, and characteristic facial abnormalities), which were not observed in any of the non22q11.2DS individuals. Also, given the prevalence of the microdeletion (1 in 4000), there is a very low likelihood for the presence of 22q11.2DS in the non22q11.2DS sample. However, future studies might consider testing all individuals for CNVs.

To address the differing gender distribution between the idiopathic ASD individuals and the other groups, we controlled for gender in the categorical fixed-effects analysis. Notably, while there is a male-biased prevalence with an estimated gender distribution of 4:1 (males to females) in idiopathic ASD [59], within 22q11.2 deletion carriers the distribution of males and females with ASD is estimated to be roughly equal [60]. Thus, the gender distribution in our sample reflects the gender distribution in the respective populations, which adds to the generalizability of our results. Furthermore, we included individuals with a relatively wide age range (i.e., from 6–25 years). Even though groups were matched in terms of their respective mean, and we corrected for linear and quadratic age effects, the nature and severity of autistic symptoms and their related neuroanatomical variability might vary across the lifespan. Hence, it will be crucial in the future to examine the multivariate correlation between the clinical ASD phenotype and neuroanatomical variability in more well defined age groups, and to characterize their association across development. Last, it is important to note that our CCA analysis was restricted to examining the multivariate association between the neuroanatomical and clinical phenotype of ASD as measured by the SRS. However, although the SRS is a well validated questionnaire that is well suited as a screening tool capturing the severity of autistic symptoms along a continuum [61], it does not by any means provide a comprehensive characterization of the complex ASD phenotype. It will therefore be crucial in the future to examine the multivariate correlation between the clinical ASD phenotype and neuroanatomical variability in larger more well defined groups using additional diagnostic measures to replicate our findings, and to better characterize the complex clinical and neurobiological phenotype across disorders. Future research may also benefit from extending the multi-dimensional phenotypic representation to include common comorbidities, such as ADHD, in the clinical phenotypic characterization, which may also provide important novel insights into the underlying mechanisms that underpin the differences we observe on the phenotypic level.

## Conclusions

Our findings indicate that the neuroanatomical correlates of ASD symptomatology in individuals with 22q11.2DS diverge from those in idiopathic ASD.

## Supplementary information

**Additional file 1: Supplementary Methods. 1.** Participant Demographics and Exclusion Criteria. **2**. Calculation of ADOS Calibrated Severity Score (CSS). **3**. MRI Data Quality Assessment, Exclusion of Scans, and Manual Edits. **4**. Canonical Correlation Analysis (CCA). **5**. Robustness of the results across feature selection algorithms. **Supplementary Tables S1.** Post-hoc multiple comparisons of means. **Supplementary Table S2.** Sample demographics after matching for age and gender. **Supplementary Table S3.** Clusters with significantly increased and decreased cortical volume (CV), surface area (SA), and cortical thickness (CT) for the main effect of 22q11.2DS. **Supplementary Table S4.** Clusters with significantly increased and decreased cortical volume (CV), surface area (SA), and cortical thickness (CT) for the main effect of ASD. **Supplementary Table S5.** Clusters with a significant 22q11.2DS-by-ASD interaction effect in cortical volume (CV) and surface area (SA). **Supplementary Figure S1.** Between-group comparison for 22q11.2DS compared to typically developing Controls. **Supplementary Figure S2.** Between-group comparison for idiopathic ASD compared to typically developing Controls. **Supplementary Figure S3.** Un-thresholded Results for the Categorical Analyses. **Supplementary Figure S4.** Effect Sizes for Categorial results. **Supplementary Figure S5.** (In)Homogeneity of Variance between idiopathic ASD and 22q11.ASD individuals. **Supplementary Figure S6.** Categorical analyses corrected for age and gender. **Supplementary Figure S7.** Schematic Overview of the Methodology behind the Canonical Correlation Analysis (CCA). **Supplementary Figure S8.** Reliability of the results across feature selection algorithms. **Supplementary Figure S9.** Boxplots for the significant 22q11.2DS-by-ASD Interaction Clusters. **Supplementary Figure S10.** Categorical 22q11.2DS-by-ASD Interaction Effect when covarying for repetitive symptoms. **Supplementary Figure S11.** Distribution of SRS subdomain and total scores across groups

## Data Availability

Further details on the data and utilized software are available upon request from the corresponding author. The full set of raw data is not currently publicly available due to ethical restrictions. However, a subset of the sample can be made available upon request.

## Abbreviations

ASD: Autism spectrum disorder
22q11.2DS: 22q11.2 deletion syndrome
CV: Cortical volume
SA: Surface area
TD: Typically developing (controls)
CCA: Canonical correlation analysis
ADHD: Attention deficit hyperactivity disorder
22q11.ASD: Individuals with 22q11.2DS and ASD
22q11.nonASD: Individuals with 22q11.2DS but no ASD
Non22q11.2DS: Individuals without the microdeletion (i.e., idiopathic ASD and TD controls)
ADI-R: Autism Diagnostic Interview-Revised
ADOS: Autism Diagnostic Observation Schedule
SRS: Social Responsiveness Scale
WASI: Wechsler Abbreviated Scale of Intelligence
CT: Cortical thickness
SAW: Social Awareness subscale of the SRS
SCG: Social Cognition subscale of the SRS
SCM: Social Communication subscale of the SRS
SM: Social Motivation subscale of the SRS
RBB: Restricted and Repetitive Behaviors subscale of the SRS
PCC: Posterior cingulate cortex
DLPFC: Dorsolateral prefrontal cortex
STG: Superior temporal gyrus
CNV: Copy number variation
