# Supplementary material for: Neuroanatomical underpinnings of autism symptomatology in carriers and non-carriers of the 22q11.2 microdeletion
Source: Mol Autism. 2020 Jun 8;11:46. doi: 10.1186/s13229-020-00356-z (PMC7282054; doi:10.1186/s13229-020-00356-z)
Supplement: Supplementary file 1 — Additional file 1: Supplementary Methods. 1. Participant Demographics and Exclusion Criteria. 2. Calculation of ADOS Calibrated Severity Score (CSS). 3. MRI Data Quality Assessment, Exclusion of Scans, and Manual Edits. 4. Canonical Correlation Analysis (CCA). 5. Robustness of the results across feature selection algorithms. Supplementary Tables S1. Post-hoc multiple comparisons of means. Supplementary Table S2. Sample demographics after matching for age and gender. Supplementary Table S3. Clusters with significantly increased and decreased cortical volume (CV), surface area (SA), and cortical thickness (CT) for the main effect of 22q11.2DS. Supplementary Table S4. Clusters with significantly increased and decreased cortical volume (CV), surface area (SA), and cortical thickness (CT) for the main effect of ASD. Supplementary Table S5. Clusters with a significant 22q11.2DS-by-ASD interaction effect in cortical volume (CV) and surface area (SA). Supplementary Figure S1. Between-group comparison for 22q11.2DS compared to typically developing Controls. Supplementary Figure S2. Between-group comparison for idiopathic ASD compared to typically developing Controls. Supplementary Figure S3. Un-thresholded Results for the Categorical Analyses. Supplementary Figure S4. Effect Sizes for Categorial results. Supplementary Figure S5. (In)Homogeneity of Variance between idiopathic ASD and 22q11.ASD individuals. Supplementary Figure S6. Categorical analyses corrected for age and gender. Supplementary Figure S7. Schematic Overview of the Methodology behind the Canonical Correlation Analysis (CCA). Supplementary Figure S8. Reliability of the results across feature selection algorithms. Supplementary Figure S9. Boxplots for the significant 22q11.2DS-by-ASD Interaction Clusters. Supplementary Figure S10. Categorical 22q11.2DS-by-ASD Interaction Effect when covarying for repetitive symptoms. Supplementary Figure S11. Distribution of SRS subdomain and total scores across groups [file 13229_2020_356_MOESM1_ESM.pdf]

Supplementary information

Supplementary Methods 2

1. Participant Demographics and Exclusion Criteria 2

2. Calculation of ADOS Calibrated Severity Score (CSS) 2

3. MRI Data Quality Assessment, Exclusion of Scans, and Manual Edits 3

4. Canonical Correlation Analysis (CCA) 4

5. Robustness of the results across feature selection algorithms 6

Supplementary Tables 7

Supplementary Tables S1 7

Supplementary Table S2 8

Supplementary Table S3 9

Supplementary Table S4 10

Supplementary Table S5 11

Supplementary Figures 12

Supplementary Figure S1. 12

Supplementary Figure S2. 13

Supplementary Figure S3. 14

Supplementary Figure S4. 15

Supplementary Figure S5 16

Supplementary Figure S6 17

Supplementary Figure S7 18

Supplementary Figure S8 19

Supplementary Figure S9. 20

Supplementary Figure S10. 21

Supplementary Figure S11. 22

References 23

Supplementary Methods

1. Participant Demographics and Exclusion Criteria

Participants were recruited and assessed at three sites: (1) the Institute of Psychiatry, Psychology, and Neuroscience (IoPPN), London, UK (n=39 including 25 microdeletion carriers and 14 typically developing (TD) controls); (2) the Semel Institute for Neuroscience and Human Behavior, University of California (UCLA), Los Angeles, US (n=39 including 25 microdeletion carriers and 14 TD controls); and (3) the Department of Child and Adolescent Psychiatry, University Hospital, Goethe University, Frankfurt, Germany (n=53 including 40 individuals with idiopathic ASD and 13 TD controls). All individuals with idiopathic ASD met the criteria for ASD (see Methods in main document), except for two females. One female scored above cut-off in the reciprocal social interaction and the repetitive behaviors domains but fell short in the communication domain. For another female, ADI-R information was unattainable, but this female scored well above the threshold on the calibrated severity score (CSS) of the ADOS (score 8 out of 10) (see Supplementary Methods 2 for further information on ADOS CSS). As our sample included few females, we decided to include both of these datasets to ensure groups were as gender-balanced as possible.

Exclusion criteria for all participants included contraindications to MRI, a medical condition or chromosomal anomaly other than 22q11.2DS, which may be associated with ASD or psychosis (e.g. tuberous sclerosis, Fragile X syndrome, or Prader-Willi syndrome). However, individuals with comorbid neuropsychiatric disorders (e.g. anxiety, depression, attention deficit hyperactivity disorder (ADHD)) were included in the 22q11.2DS and idiopathic ASD groups, as these are common comorbid features of both conditions. All participants, and accompanying parents for those under 18 years of age, gave informed written consent in accordance with ethics approval by the Ethics Committee of the Faculty of Medicine of the Goethe University Frankfurt, the National Research Ethics Service (NRES) Committee South Central (study reference: 12/SC/0576), or the UCLA Institutional Review Board (IRB).

2. Calculation of ADOS Calibrated Severity Score (CSS)

To allow comparability of ADOS total severity scores across different ADOS versions and modules, we computed ADOS Calibrated Severity Scores (CSS). The majority of individuals received Module 3 or 4 of ADOS-G in London and Los Angeles and of ADOS-2 in Frankfurt. While ADOS-2 total severity scores were calculated according to the ADOS-2 Manual (1), the calculation of the CSS for Module 4 was conducted according to Hus & Lord (2014) (2). As for ADOS-G, raw scores were initially mapped onto ADOS-2 raw scores (1-3) and subsequently CSS was calculated accordingly. One participant in the 22q11.nonASD group received an ADOS-G Module 2, while one participant in the idiopathic ASD group received an ADOS-2 Module 1. The calculation of ADOS CSS for Modules 1 and 2 is analogue to Module 3 (1).

3. MRI Data Quality Assessment, Exclusion of Scans, and Manual Edits

The FreeSurfer derived surface models were visually inspected for reconstruction errors, and the quality of each scan was rated using one of the following three options: (1) accept as is (no visible reconstruction errors or artefacts), (2) prescribe manual edits (visible reconstruction errors in either pial or white matter surface (or both) that might be recoverable using manual edits), or (3) exclude (gross anatomical abnormalities or severe acquisition artefacts). Manual edits were performed by making changes to either the pial (i.e. grey matter) or white matter surface, or both. Following manual editing, images were (re-)pre-processed and re-assessed for reconstruction errors.

22q11.2DS and controls from IoPPN and UCLA

For a previous study published by our group, we completed manual edits. In this study, we started off with 172 scans in total, acquired across two sites (n=81 at IoPPN, n=91 at UCLA), for which FreeSurfer pre-processing was performed. The resulting surface models were then visually inspected for reconstruction errors and scans were either (1) accepted 'as is' (n=112 out of 172 or 65%), (2) rejected 'as is' (n=34 out of 172 or 20%) – mostly due to severe (motion) artefacts, segmentation errors, or the existence of extra-brain tissue (e.g. dura) that precluded a successful FreeSurfer reconstruction, or (3) referred for manual editing (n=26 out of 172 or 15%) in case of smaller 'local' reconstruction errors. Out of the 26 scans that were re-pre-processed, 19 surface reconstructions (73%) did not improve significantly following manual editing and were subsequently excluded from the statistical analysis. This meant that a total of 53 scans (31%) were excluded overall. This overall dropout was approximately equal across sites, with 28 out of 91 scans being excluded from UCLA (i.e. 30.77% including n=15 controls, n=4 22q11.ASD, and n=9 22q11.nonASD individuals), and 25 out of 81 scans being excluded from the IoPPN (i.e. 30.86% including n=12 controls, n=6 22q11.ASD, and n=7 22q11.nonASD individuals). In terms of diagnostic categories, we excluded a total of 27 out of 84 controls (32%), and 26 out of 88 22q11.2DS individuals (29.5%). Dropout rates for cases and controls were therefore very closely matched. Moreover, out of 81 individuals with available ADI-R data, we excluded 10 out of 40 (i.e. 25%) 22q11.ASD individuals, and 16 out of 41 (39%) 22q11.nonASD individuals. However, the difference in the proportion of excluded ASD and nonASD individuals was not statistically significant (χ^2=1.2403, df=1, p=0.2654). Thus, although the dropout of ~30% is relatively high, we are confident that there is no systematic sampling bias that could have influenced our results in terms of the 22q11.2DS groups and their matched controls.

For the purpose of the present study, 7 out of the original n=62 with 22q11.2DS did not have ADI-R information, and a further 5 were too old to be matched with the idiopathic ASD group so were further excluded. Of the 57 controls, we selected n=14 to match our overall groups.

Idiopathic ASD individuals and controls from Frankfurt

The scans acquired in Frankfurt were selected from an ongoing study examining the neuroanatomical underpinnings of autism in children and adolescents with idiopathic ASD relative to TD controls based on participant's age, gender, and IQ. Here, quality assessments and manual edits were conducted using the same stringent criteria as outlined for the 22q11.2DS group.

4. Canonical Correlation Analysis (CCA)

CCA aims to describe the linear relationship between a set of $n \times p$ predictor variables *X,* and $n \times q$ outcome measures Y, where *n* indicates the number of participants. Initially, CCA estimates two parameter vectors w_x and w_y so that the correlation ρ between the linear combinations $\hat{X} = w_x^T X$ and $\hat{Y} = w_y^T Y$ is maximised, i.e.

$$\rho = \mathrm{corr}(\hat{X}, \hat{Y}) = \max_{w_x, w_y} \ \mathrm{corr}(w_x^T X, w_y^T Y).$$

The resulting predicted variables $\hat{X}_1$ and $\hat{Y}_1$ are the first pair of *canonical variates*, and their correlation ρ_1 is the first *canonical correlation*. Similar to Principle Component Analysis (PCA), the 2nd set of parameter estimates maximizing ρ is then derived subject to the constraint of being uncorrelated with the first pair of canonical variates. This procedure may be continued up to i times, where $i = \min\{p, q\}$, resulting in maximally i canonical variate pairs (see Supplementary Figure S7).

Classical CCA assumes that the number of variables in *X* and *Y* is lower than the number of samples (i.e. $n \leq \max\,(p, q)$). We therefore initially reduced the large number of neuroanatomical features (*n*=204) to a smaller subset of clinically relevant features using six different variable selection approaches: 1) based on bivariate *Pearson's correlation* coefficients between *X* and *Y* features, selecting neuroanatomical features that displayed at least one significant correlation with at least one of the SRS subdomains (*p*<0.05; two-tailed); 2) stepwise regression with bidirectional variable inclusion and Akaike Information Criterion (AIC)-based model selection (4); 3) feature selection using the *Boruta* algorithm that is built on a Random Forest regression algorithm, and iteratively removes features that are statistically proven to be less relevant than random probes using an adjusted *p*-value < 0.05 (5); 4) using *Least Absolute Shrinkage and Selection Operator* (LASSO) regression implemented in the R 'glmnet' package (6), retaining features with a permutation-based variable importance larger than zero; 5) permutation importance (PIMP)-based variable selection (7)for Random Forest classifiers, selecting variables with a *p*-value < 0.05; and 6) using the *Novel Testing Approach* (NTA) by Janitza et al. (2015), which provides *p*-values for the cross-validated permutation variable importance measures (*p*<0.05) (8). We selected these feature selection approaches to sample results across *filter* methods (e.g. Pearson's correlation), *wrapper* methods (e.g. stepwise regression), and *embedded* methods (e.g. glmnet). Moreover, these methods provided a subset of features that was sufficiently large to be

meaningfully compared between groups, yet meeting the constraint of $n \leq \max(p, q)$. The neuroanatomical subsets highlighted by the different feature selection approaches are shown in Supplementary Figure S8. We based our analysis on the feature selection algorithm providing the largest subset of clinically relevant neuroanatomical features, which was the stepwise regression procedure highlighting a set of *p*=63 measures overall.

CCA model fitting and evaluation was then performed as outlined in the Methods Section of the main document (see pages 10-12). To compare the factor loadings structure of clinical and neuroanatomical canonical variates across groups, we applied the *forward model* of the form $x = \mathbf{\Lambda}\hat{x} + \varepsilon$ (9) where

$$\mathbf{\Lambda} \propto X^T X \, \mathbf{W} = X^T \hat{X} = cov(X, \hat{X})$$

to data coming from carriers and non-carriers of the 22q11.2 microdeletion, resulting in a set of group-specific clinical and neuroanatomical loadings matrices. The *Tucker's congruence coefficient* (10) was used to establish the degree of factor similarity between groups. The congruence coefficient constitutes the cosine of the angle between two vectors (i.e. clinical and/or neuroanatomical loadings for 22q11.2DS and non22q11.2DS individuals on each canonical variate), and can be interpreted as a standardized measure of proportionality of elements in both vectors. It is formalized as

$$\phi(\lambda_{22q}, \lambda_{non22q}) = \frac{\sum \lambda_{22q} \lambda_{non22q}}{\sqrt{\sum \lambda_{22q}^2 \sum \lambda_{non22q}^2}},$$

where λ_{22q} and λ_{non22q} denote the loadings of variables/features in carriers and non-carriers of the 22q11.2 microdeletion. A congruence coefficient in the range of [0.85-0.94] corresponds to a *fair* similarity, while a value >0.95 implies that the structure of two factors might be considered *equal* (11).

To establish the reliability of our findings, we repeated the CCA and the comparison of neuroanatomical and clinical factor loadings between groups across the six different feature selection approaches. Here, we initially compared neuroanatomical features across selection approaches based on a statistical difference in factor loadings between groups (see *Fisher's Z* test, *p*<0.05, one-tailed). For each neuroanatomical feature, we then derived a *confidence score* indicating the level of confidence associated with the hypothesis of a between-group difference in loadings across feature selection algorithms. This confidence score took into account (1) the number of times a feature was highlighted as being clinically relevant across feature selection algorithms (e.g. feature selected by 2 out of 6 selection approaches), and (2) the number of times a feature was highlighted as displaying a statistically significant between-group difference in factor loading across canonical variates, relative to the number of times a feature was selected as being clinically relevant (e.g. a feature loading was significantly different between groups in 2 out of 3 selections). The confidence score was simply the product of (1) and (2), yielding confidence scores ranging between 0 and 2. Last, we examined the *Tucker's congruence coefficient* for each set of clinical and neuroanatomical

loadings in carriers and non-carriers of the 22q11.2 microdeletion derived by the different feature selection algorithms.

5. Robustness of the results across feature selection algorithms

Supplementary Figure S8 shows the individual subsets of neuroanatomical features that were highlighted as being clinically relevant by the six different feature selection algorithms. As expected, the different feature selection approaches resulted in different, overlapping sets of neuroanatomical features with some neuroanatomical features being highlighted as clinical relevant by all approaches (see Supplementary Figure S8A). Overall, the stepwise regression approach was the most inclusive, selecting a total number of 63 features in total, followed by the *NTA* approach (*n*=51 features), PIMP (*n*=35), *glmnet* (*n*=21), significant *Pearson's* correlation coefficients (*n*=21), and *Boruta* (*n*=16). We therefore based the analysis presented within the main manuscript on the *n*=63 features highlighted by the stepwise regression. Strongest agreement (i.e. level of confidence) on a significant difference in neuroanatomical factor loadings between carriers and non-carriers of the 22q11.2 microdeletion was observed in the volume of the left superior parietal lobe, which was highlighted by all feature selection approaches as clinically relevant, and being significantly different between groups (confidence score = 1), followed the right pars orbitalis based on measures of CT, which was significantly different in 4 our of 6 selections (confidence score = 0.67) (see Supplementary Figure S8A). Moreover, across all feature selection approaches, we observed a high degree of congruence in the clinical factor structure with a Tucker's coefficient of >0.94 (see Supplementary Figure S8B), but a low level of congruence (i.e. Tucker's coefficient <0.94) in neuroanatomical loadings between carriers and non-carriers of the 22q11.2 microdeletion (see Supplementary Figure S8C). The results across feature selection approaches thus converge in suggesting across that carriers and non-carriers of the 22q11.2 microdeletion display a very similar clinical factor structure, which is underpinned by different neuroanatomical substrates.

Supplementary Tables

Supplementary Tables S1. Post-hoc multiple comparisons of means

Full-scale IQ: F(3)=21.01, *p*<0.001

TD controls > ASD > 22q11.ASD = 22q11.nonASD

	22q11.ASD	22q11.nonASD	TD controls	ASD
**22q11.ASD**		0.619	<0.001	<0.001
**22q11.nonASD**	0.619		<0.001	0.020
**TD controls**	<0.001	<0.001		0.057
**ASD**	<0.001	0.020	0.057	

Note: Scheffé Test – signicance values between groups.

Total Cortical Volume: F(3)=10.56, *p*<0.001

ASD = TD controls > 22q11.ASD = 22q11.nonASD

	22q11.ASD	22q11.nonASD	TD controls	ASD
**22q11.ASD**		0.861	0.048	0.003
**22q11.nonASD**	0.861		0.003	<0.001
**TD controls**	0.048	0.003		0.701
**ASD**	0.003	<0.001	0.701	

Note: Scheffé Test – signicance values between groups.

Total Surface Area: F(3)=12.41, *p*<0.001

ASD = TD controls > 22q11.ASD = 22q11.nonASD

	22q11.ASD	22q11.nonASD	TD controls	ASD
**22q11.ASD**		0.852	0.011	0.001
**22q11.nonASD**	0.852		<0.001	<0.001
**TD controls**	0.011	<0.001		0.883
**ASD**	0.001	<0.001	0.883	

Note: Scheffé Test – signicance values between groups.

Mean Cortical Thickness: F(3)=3.74, *p*<0.05

22q11.ASD = 22q11.nonASD > ASD = TD controls

	22q11.ASD	22q11.nonASD	TD controls	ASD
**22q11.ASD**		0.988	0.218	0.194
**22q11.nonASD**	0.988		0.101	0.088
**TD controls**	0.218	0.101		0.100
**ASD**	0.194	0.088	0.100	

Note: Scheffé Test – signicance values between groups.

Supplementary Table S2. Sample demographics after matching for age and gender

	22q11.nonASD	22q11.ASD	ASD	Controls
N	11	18	25	30
Age (mean)	14.27	14.28	14.28	13.90
Females (%)	36%	33%	33%	32%

Supplementary Table S3. Clusters with significantly increased and decreased cortical volume (CV), surface area (SA), and cortical thickness (CT) for the main effect of 22q11.2DS

Contrast	Cluster	Region Labels	Hemisphere	BA	Vertices	Talairach			t_{max}	$p_{cluster}$
						x	y	z		
**Cortical Volume**										
**22q11.2DS > non22q11.2DS**										
	1	Insula, pars opercularis, postcentral gyrus, precentral gyrus, supramarginal gyrus	R	1-4, 6, 13, 40, 43- 44	10963	32	10	11	5.26	1.97×10^{-5}
	2	Insula, lateral orbital frontal cortex, pars orbitalis, supramarginal gyrus	L	6, 13, 40, 43-45, 47	7242	-35	-18	1	5.25	1.97×10^{-5}
	3	Postcentral gyrus, precentral gyrus	L	1-4	4035	-61	-8	26	4.31	1.97×10^{-5}
	4	Precentral gyrus, superior frontal gyrus	R	4, 6, 8-9	3404	20	15	51	4.14	1.97×10^{-5}
	5	Middle temporal gyrus	L	21, 38	1103	-49	-6	-25	4.46	8.18×10^{-5}
	6	Medial orbital frontal cortex	R	11, 25	909	8	18	-18	3.92	4.26×10^{-4}
	7	Superior frontal gyrus	L	6, 8-9	1265	-19	33	43	3.32	6.16×10^{-4}
	8	Medial orbital frontal cortex	L	11, 25	659	-8	16	-15	3.74	2.64×10^{-3}
	9	Lateral orbital frontal cortex	R	46	701	31	33	-8	3.14	6.06×10^{-3}
	10	Postcentral gyrus, precentral gyrus	L	1-4, 43	901	-57	0	17	3.62	1.37×10^{-2}
**22q11.2DS < non22q11.2DS**										
	1	Cuneus cortex, lingual gyrus, parahippocampal gyrus, pericalcarine cortex, precuneus cortex, superior parietal cortex	L	7, 17-19, 36	9341	-19	-43	-5	-5.90	1.97×10^{-5}
	2	Cuneus cortex, isthmus cingulate cortex, lingual gyrus, precuneus cortex, superior parietal cortex	R	7, 17, 19, 30	4061	18	-53	21	-4.52	1.97×10^{-5}
	3	Lingual gyrus, parahippocampal gyrus	R	19, 36	1926	21	-39	-6	-4.43	1.98×10^{-5}
	4	Fusiform gyrus, lateral occipital cortex	L	19, 37	1839	-31	-81	-7	-4.59	2.09×10^{-5}
	5	Postcentral gyrus, precentral gyrus	R	1-3, 5	1927	4	-25	62	-3.54	5.62×10^{-4}
	6	Caudal anterior cingulate cortex	L	23-24, 33	760	-6	25	17	-3.10	1.58×10^{-3}
	7	Paracentral lobule	L	4, 6	1291	-7	-33	53	-4.12	2.99×10^{-3}
	8	Fusiform gyrus, lateral occipital cortex	R	18-19, 37	937	29	-79	-3	-4.37	3.41×10^{-3}
	9	Caudal anterior cingulate cortex	R	24, 33	1098	9	30	11	-3.82	4.42×10^{-3}
**Surface Area**										
**22q11.2DS > non22q11.2DS**										
	1	Caudal middle frontal gyrus, insula, pars opercularis, postcentral gyrus, precentral gyrus	R	1-4, 6, 13, 43-44	11464	37	-10	-8	5.87	1.26×10^{-5}
	2	Caudal middle frontal gyrus, insula, pars opercularis, postcentral gyrus, precentral gyrus, superior temporal gyrus, supramarginal gyrus	L	1-4, 6, 13, 40-41, 43-44	11109	-34	-24	20	4.25	1.26×10^{-5}
	3	Precentral gyrus, superior frontal gyrus	R	4, 6, 8-9	5760	22	7	54	5.01	1.26×10^{-5}
	4	Insula, lateral orbital frontal cortex, pars opercularis, pars orbitalis, pars triangularis	L	13, 44-45, 47	3411	-40	26	-13	4.22	1.28×10^{-5}
	5	Superior frontal gyrus	L	4, 6, 8-9	3952	-18	0	61	3.73	1.49×10^{-5}
	6	Medial orbital frontal cortex	R	11, 25	1134	8	25	-10	3.66	1.42×10^{-4}
	7	Posterior cingulate cortex	L	23	442	-4	-11	28	4.16	1.63×10^{-4}
**22q11.2DS < non22q11.2DS**										
	1	Cuneus cortex, fusiform gyrus, inferior parietal cortex, lateral occipital cortex, lingual gyrus, parahippocampal gyrus, pericalcarine cortex, precuneus cortex, superior parietal cortex	L	7, 17-19, 30-31, 36-37, 39	18715	-11	-73	22	-6.41	1.26×10^{-5}
	2	Cuneus cortex, fusiform gyrus, lateral occipital cortex, lingual gyrus, parahippocampal gyrus, pericalcarine cortex, precuneus cortex, superior parietal cortex	R	7, 17-19, 30-31, 36-37	15034	25	-72	-1	-5.65	1.26×10^{-5}
	3	Inferior temporal gyrus, temporal pole	L	20, 38	1463	-30	-1	-24	-4.28	1.26×10^{-5}
	4	Caudal anterior cingulate cortex, superior frontal gyrus	L	6, 9-10, 23, 32	2997	-12	25	22	-4.16	1.66×10^{-5}
	5	Fusiform gyrus, inferior temporal gyrus	R	20, 37	2632	39	-1	-32	-4.74	5.89×10^{-5}
	6	Paracentral lobule, superior frontal gyrus	R	4, 6, 32	2072	8	-24	54	-3.94	2.48×10^{-4}
	7	Postcentral gyrus, precentral gyrus	R	1-4	1857	23	-27	65	-3.92	1.80×10^{-3}
	8	Caudal anterior cingulate cortex, rostral anterior cingulate cortex, superior frontal gyrus	R	6, 24, 32-33	1372	11	33	14	-3.87	1.38×10^{-2}
	9	Paracentral lobule	L	4	1103	-13	-21	35	-4.02	2.32×10^{-2}
	10	Postcentral gyrus, precentral gyrus	L	1-3	1258	-29	-25	46	-3.10	3.89×10^{-2}
**Cortical Thickness**										
**22q11.2DS > non22q11.2DS**										
	1	Postcentral gyrus	R	1-3	947	51	-11	29	4.04	1.64×10^{-2}
	2	Lingual gyrus	L	17, 19	831	-5	-81	2	4.15	1.80×10^{-2}
	3	Pericalcarine cortex	R	17, 19	767	12	-86	12	4.13	2.26×10^{-2}
	4	Supramarginal gyrus	L	40	759	-46	-32	34	3.83	4.02×10^{-2}
**22q11.2DS < non22q11.2DS**										
	1	Superior temporal gyrus, transverse temporal cortex	R	22, 38, 41-42	3215	50	-11	-5	-5.01	2.18×10^{-5}
	2	Superior temporal gyrus, transverse temporal cortex	L	22, 38, 41-42	2933	-51	-13	2	-5.06	2.18×10^{-5}
	3	Posterior cingulate cortex	R	23-24	1042	4	-8	34	-4.52	2.19×10^{-5}
	4	Posterior cingulate cortex	L	23-24	846	-5	12	27	-3.74	3.73×10^{-5}
	5	Parahippocampal gyrus	L	36	976	-21	-29	-14	-4.71	8.65×10^{-5}
	6	Parahippocampal gyrus	R	36	645	21	-30	-10	-4.03	4.90×10^{-2}

Note: Hemisphere: *L*: Left, *R*: Right; *BA*: approxminate Brodmann area(s); Vertices: number of vertices within the cluster; *t*max: maximum *t*-statistic within the cluster; *p*-cluster: cluster-corrected *p*-value.

Supplementary Table S4. Clusters with significantly increased and decreased cortical volume (CV), surface area (SA), and cortical thickness (CT) for the main effect of ASD

Contrast	Cluster	Region Labels	Hemisphere	BA	Vertices	Talairach x	Talairach y	Talairach z	t_{max}	$p_{cluster}$
**Cortical Volume**										
**ASD > nonASD**										
	1	Insula, superior temporal gyrus, transverse temporal cortex	L	13, 22, 41	2237	-41	-25	3	3.85	2.32×10^{-5}
	2	Inferior parietal cortex	R	39	816	42	-64	34	3.09	4.83×10^{-2}
**ASD < nonASD**										
	1	Entorhinal cortex	L	36	864	-24	-5	-27	-4.01	1.97×10^{-5}
**Surface Area**										
**ASD > nonASD**										
	1	Insula, superior temporal gyrus	L	13, 22, 43	1613	-42	-24	-1	4.99	6.72×10^{-4}
	2	Banks superior temporal sulcus, superior temporal gyrus , supramarginal gyrus	L	40-42	2102	-58	-48	21	3.72	7.30×10^{-3}
	3	Fusiform gyrus, lingual gyrus, parahippocampal gyrus	L	19, 36-37	1175	-33	-33	-12	3.53	4.74×10^{-2}
**ASD < nonASD**										
	1	Enthorinal cortex, fusiform gyrus	L	36-38	1349	-24	-9	-25	-3.72	1.26×10^{-5}
**Cortical Thickness**										
**ASD > nonASD**										
	1	Isthmus cingulate cortex	R	29	689	19	-47	5	3.50	5.33×10^{-3}
	2	Superior temporal gyrus	R	22, 41-42	917	49	-25	0	3.36	1.56×10^{-2}

Note: Hemisphere: *L*: Left, *R*: Right; *BA*: approxminate Brodmann area(s); Vertices: number of vertices within the cluster; *t*max: maximum *t*-statistic within the cluster; *p*-cluster: cluster-corrected *p*-value.

Supplementary Table S5. Clusters with a significant 22q11.2DS-by-ASD interaction effect in cortical volume (CV) and surface area (SA)

Contrast	Cluster	Region Labels	Hemisphere	BA	Vertices	Talairach			t_{max}	$p_{cluster}$
						x	y	z		
**Cortical Volume**										
**22q11.2DS*ASD**										
	1	Pars opercularis, pars triangularis	L	44-46	1302	-43	25	13	3.84	3.09×10^{-4}
	2	Precentral gyrus	R	4	1445	41	-8	32	3.12	7.39×10^{-3}
**Surface Area**										
**22q11.2DS*ASD**										
	1	Posterior cingulate cortex	L	23-24, 31	1782	-4	-12	28	3.93	1.26×10^{-5}
	2	Pars opercularis, pars triangularis, precentral gyrus, rostral middle frontal gyrus	L	44-46	3786	-53	22	12	4.38	2.32×10^{-5}

Note: Hemisphere: *L*: Left, *R*: Right; *BA*: approxminate Brodmann area(s); Vertices: number of vertices within the cluster; *t*max: maximum *t*-statistic within the cluster; *p*-cluster: cluster-corrected *p*-value.

Supplementary Figures

Supplementary Figure S1. *Between-group comparison for 22q11.2DS compared to typically developing Controls*

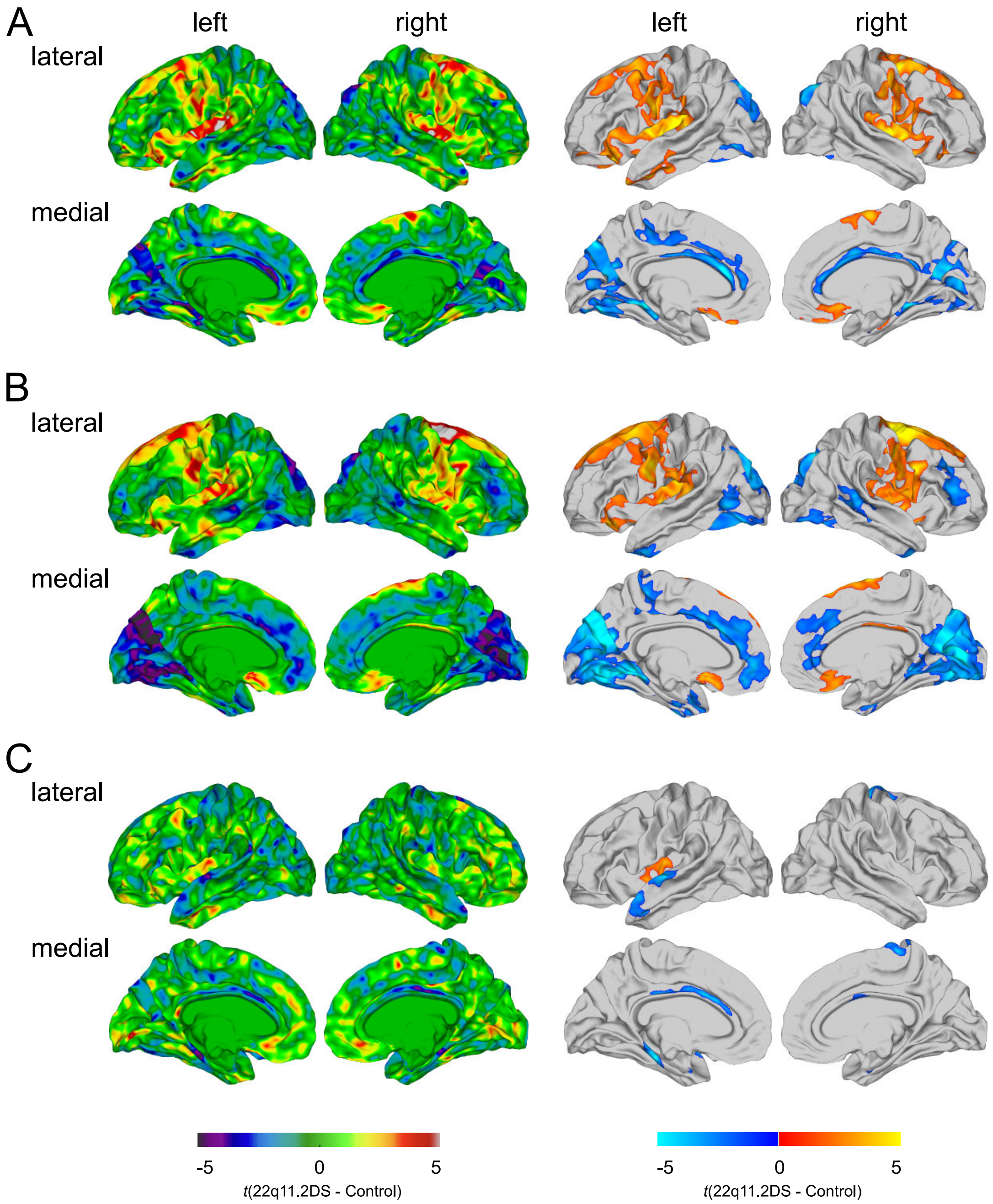

Significant differences in cortical volume (CV; A), surface area (SA; B), and cortical thickness (CT; C) in all individuals with 22q11.2DS compared to typically developing (TD) controls. The left panel shows the un-thresholded *t*-maps where increases in 22q11.2DS relative to TD controls are indicated in yellow to red, and decreases in blue to cyan. The right panel shows the random-field-theory (RFT)-based cluster-corrected ($p<0.05$, two-tailed) difference maps indicating significant increases (marked in red to yellow) and decreases (marked in blue) following correction for multiple comparisons.

Supplementary Figure S2. *Between-group comparison for idiopathic ASD compared to typically developing Controls*

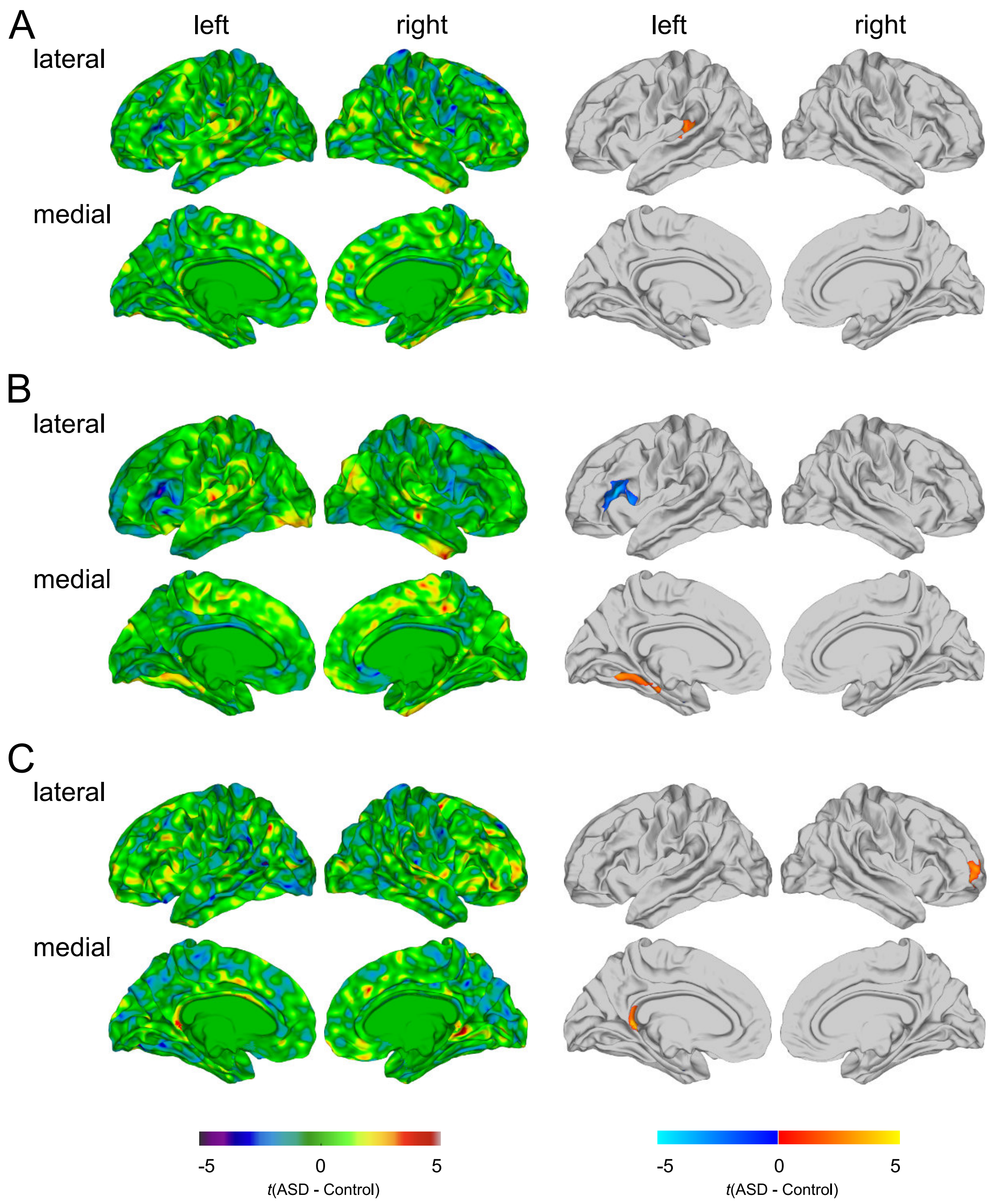

Significant differences in cortical volume (CV; A), surface area (SA; B), and cortical thickness (CT; C) in individuals with idiopathic ASD compared to typically developing (TD) controls. The left panel shows the un-thresholded *t*-maps where increases in ASD relative to TD controls are indicated in yellow to red, and decreases in blue to cyan. The right panel shows the random-field-theory (RFT)-based cluster-corrected ($p<0.05$, two-tailed) difference maps indicating significant increases (marked in red to yellow) and decreases (marked in blue) following correction for multiple comparisons.

Supplementary Figure S3. *Un-thresholded Results for the Categorical Analyses*

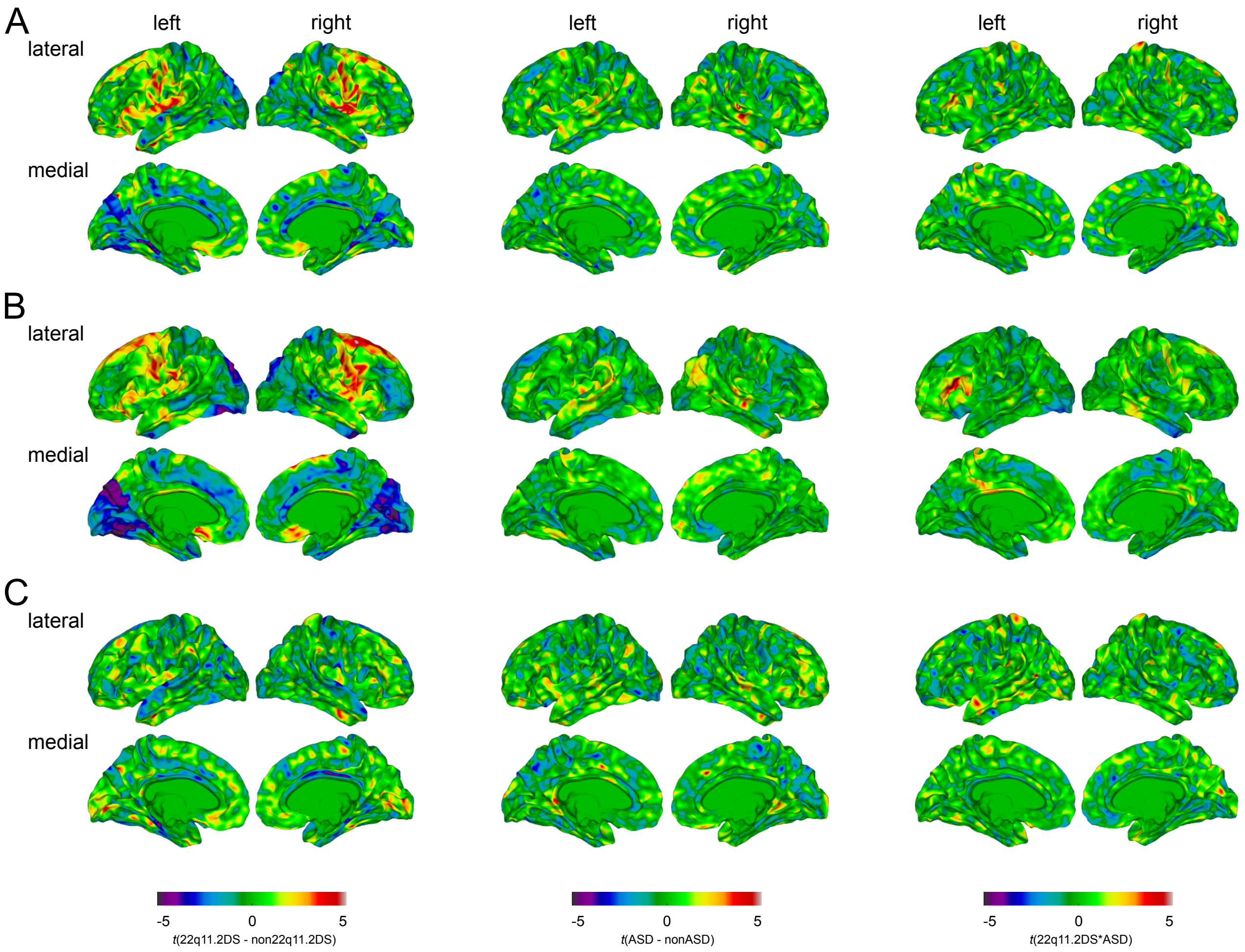

Significant differences in cortical volume (CV; A), surface area (SA; B), and cortical thickness (CT; C) for the main effect of 22q11.2DS (left panel; i.e. all individuals with 22q11.2DS compared to typically developing (TD) controls and idiopathic ASD), the main effect of ASD (middle panel; i.e. all individuals with ASD symptomatology (22q11.ASD and idiopathic ASD) compared to nonASD individuals (TD controls and 22q11.nonASD), and for the 22q11.2DS-by-ASD interaction (right panel). Displayed are the un-thresholded *t*-maps where increased parameter estimates in 22q11.2DS, respectively ASD, are marked in red to yellow, and decreased parameters are marked in blue to cyan.

Supplementary Figure S4. *Effect Sizes for Categorial results*

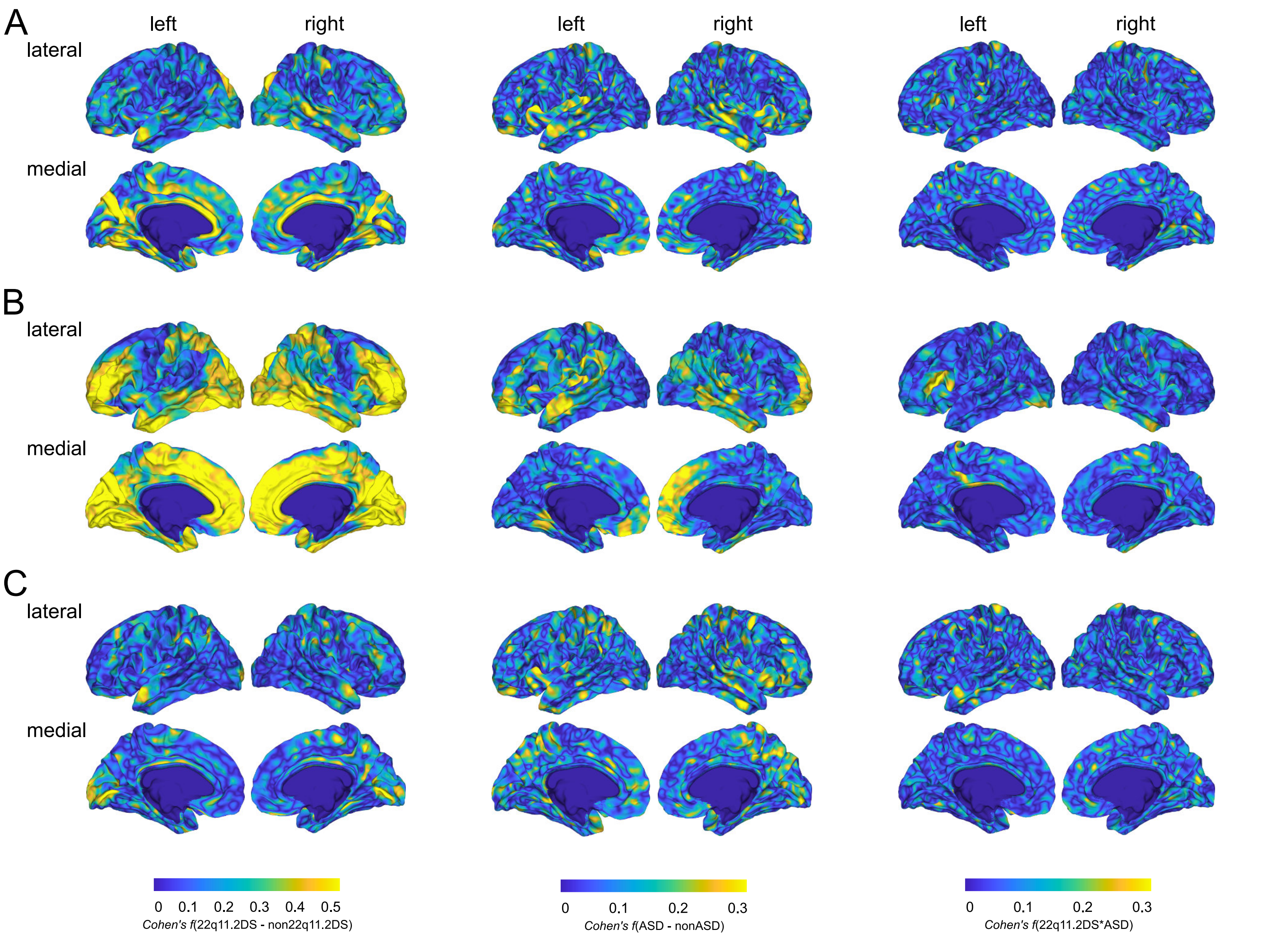

Effect sizes (Cohen's f) for the main effect of 22q11.2DS (left panel), the main effect of ASD (middle panel), and for the 22q11.2DS-by-ASD interaction term (right panel). Effect sizes for vertex-wise estimates of cortical volume (CV) are shown in (A), for surface area (SA) in (B), and cortical thickness (CT) in (C).

Supplementary Figure S5. *(In)Homogeneity of Variance between idiopathic ASD and 22q11.ASD individuals*

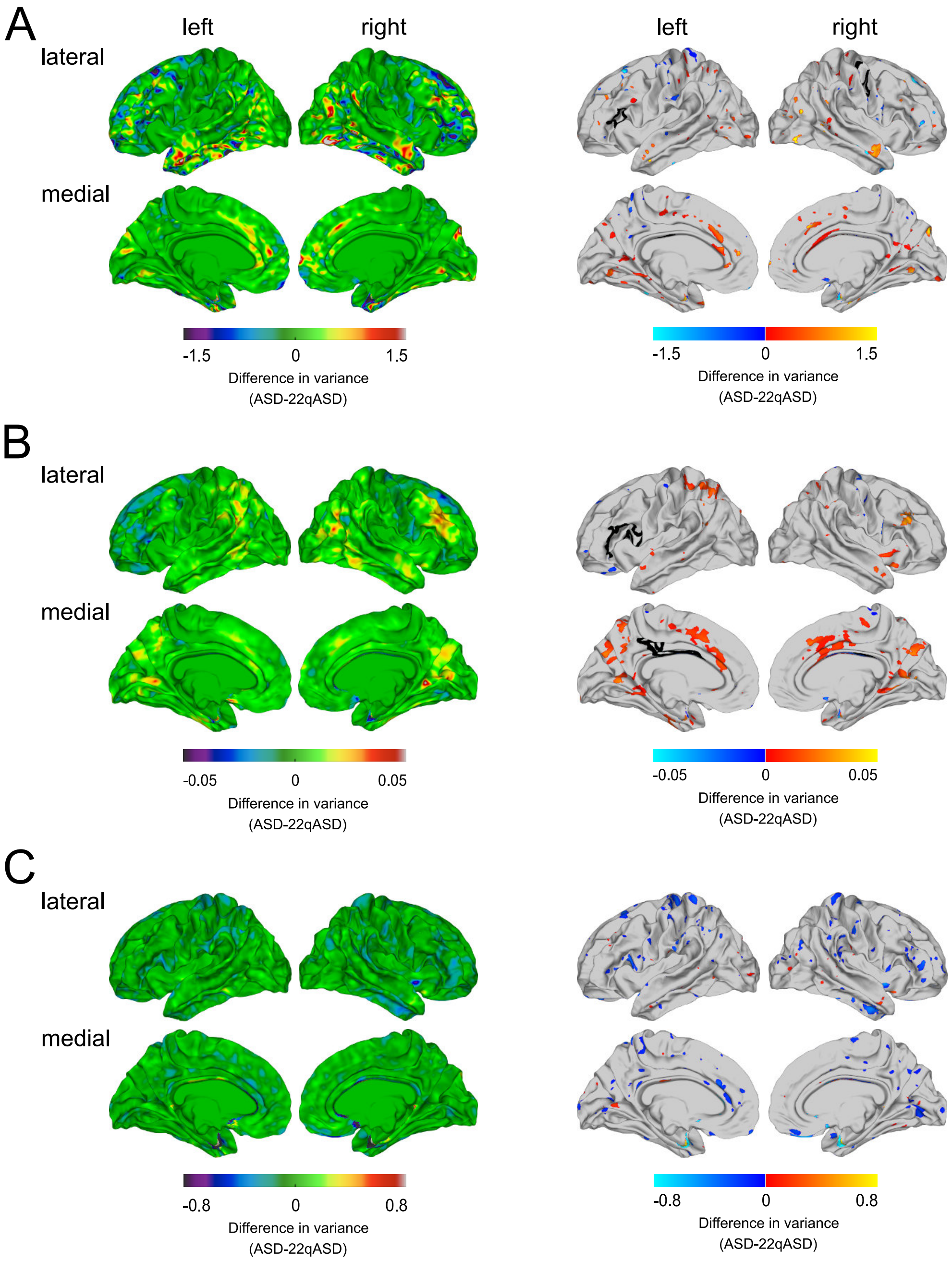

The left panel shows vertex-wise differences in variance between individuals with idiopathic ASD (ASD) and individuals with the 22q11.2 microdeletion and ASD (22qASD) for cortical volume (CV; A), surface area (SA; B), and cortical thickness (CT; C). The right panel shows vertices with a significant difference in variance as assessed using Levene's test of homogeneity of variance following a FDR correction for multiple comparisons. Significant increases in variance in ASD compared to 22qASD are marked in red to yellow, and decreased variances are marked in blue. The regions-of-interest marked by black lines correspond to the clusters with a significant 22q11.2DS-by-ASD interaction effect as resulting from the categorical analyses.

Supplementary Figure S6. *Categorical analyses corrected for age and gender*

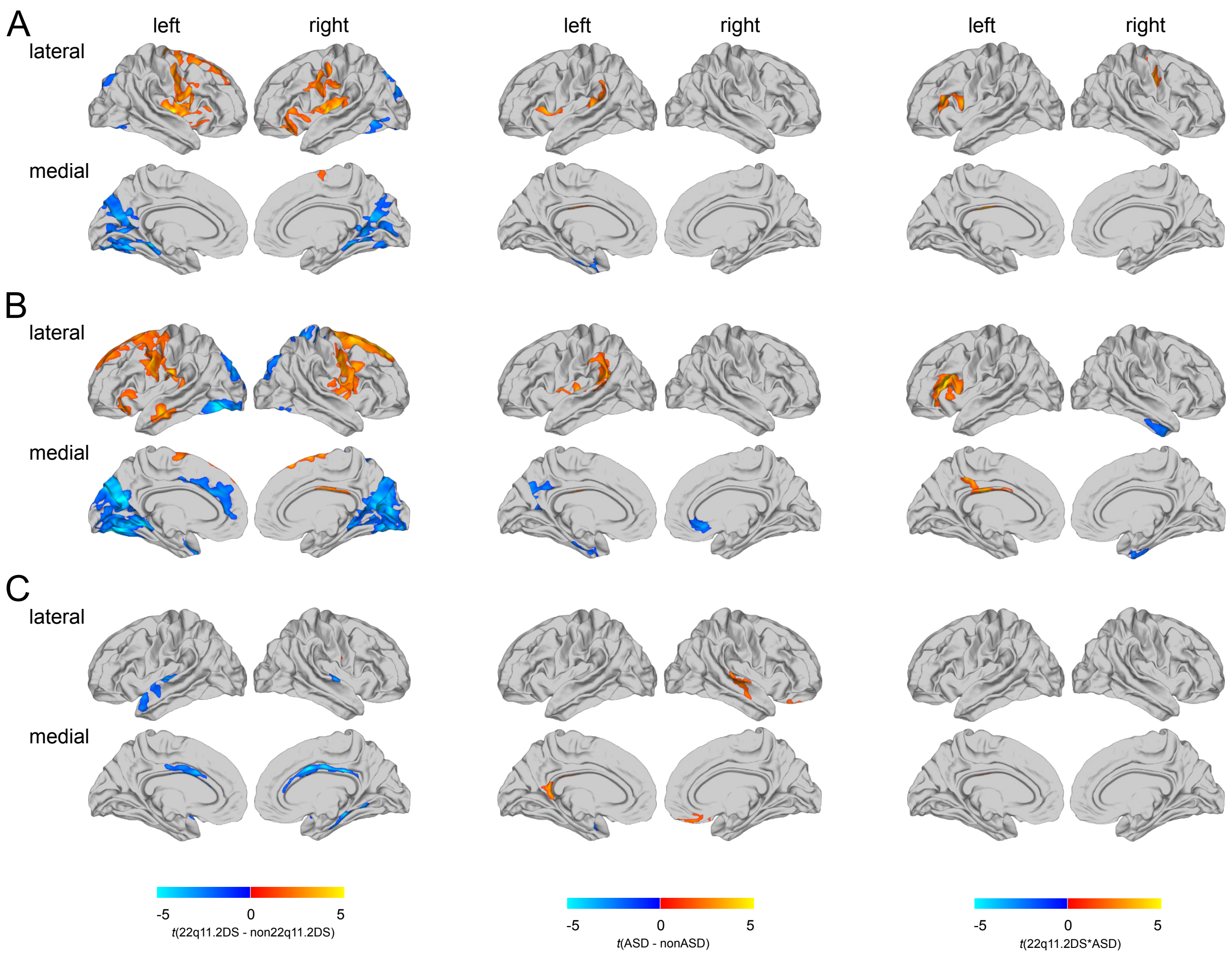

Significant differences in cortical volume (CV; A), surface area (SA; B), and cortical thickness (CT; C) for the main effect of 22q11.2DS (left panel; i.e. all individuals with 22q11.2DS compared to typically developing (TD) controls and idiopathic ASD), the main effect of ASD (middle panel; i.e. all individuals with ASD symptomatology (22q11.ASD and idiopathic ASD) compared to nonASD individuals (TD controls and 22q11.nonASD), and for the 22q11.2DS-by-ASD interaction (right panel). Displayed are the random field theory (RFT)-based cluster corrected ($p<0.05$, 2-tailed) difference maps following multiple comparisons, where increased parameter estimates in 22q11.2DS (or ASD) are marked in red to yellow, and decreased parameters are marked in blue to cyan.

Supplementary Figure S7. *Schematic Overview of the Methodology behind the Canonical Correlation Analysis (CCA)*

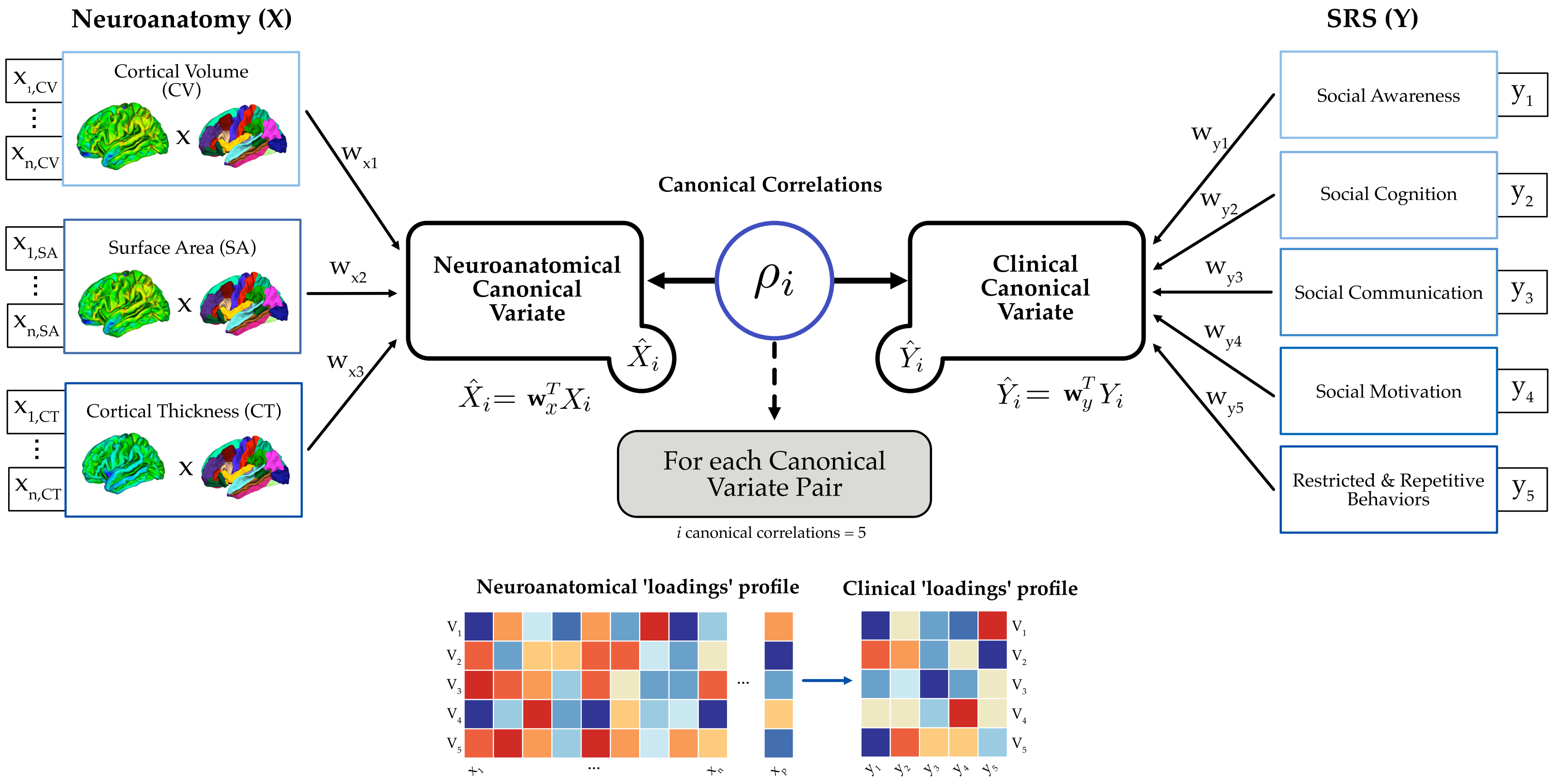

CCA was used to examine the relationship between neuroanatomical variability in cortical volume (CV), surface area (SA), and cortical thickness (CT) as predictors (*X;* left panel), and the five Social Responsiveness Scale (SRS) subdomain scores in (1) social awareness (SAW), (2) social cognition (SCG), (3) social communication (SCM), (4) social motivation (SM), and (5) restricted and repetitive behaviors (RRB) as clinical outcomes (*Y*; right panel). CCA estimates two parameter vectors w_x and w_y so that the correlation ρ between the linear combinations $\hat{X} = w_x^T X$ and $\hat{Y} = w_y^T Y$ is maximised. The resulting predicted variables $\hat{X}_1$ and $\hat{Y}_1$ are the first pair of canonical variates (*V*s), and their correlation ρ_1 is the first canonical correlation. The 2nd set of parameter estimates maximising ρ is then derived subject to the constraint of being uncorrelated with the first pair of canonical variates. This procedure may be continued up to i times, where $i = \min\{p, q\}$, resulting in maximally i canonical variate pairs.

Supplementary Figure S8. *Reliability of the results across feature selection algorithms*

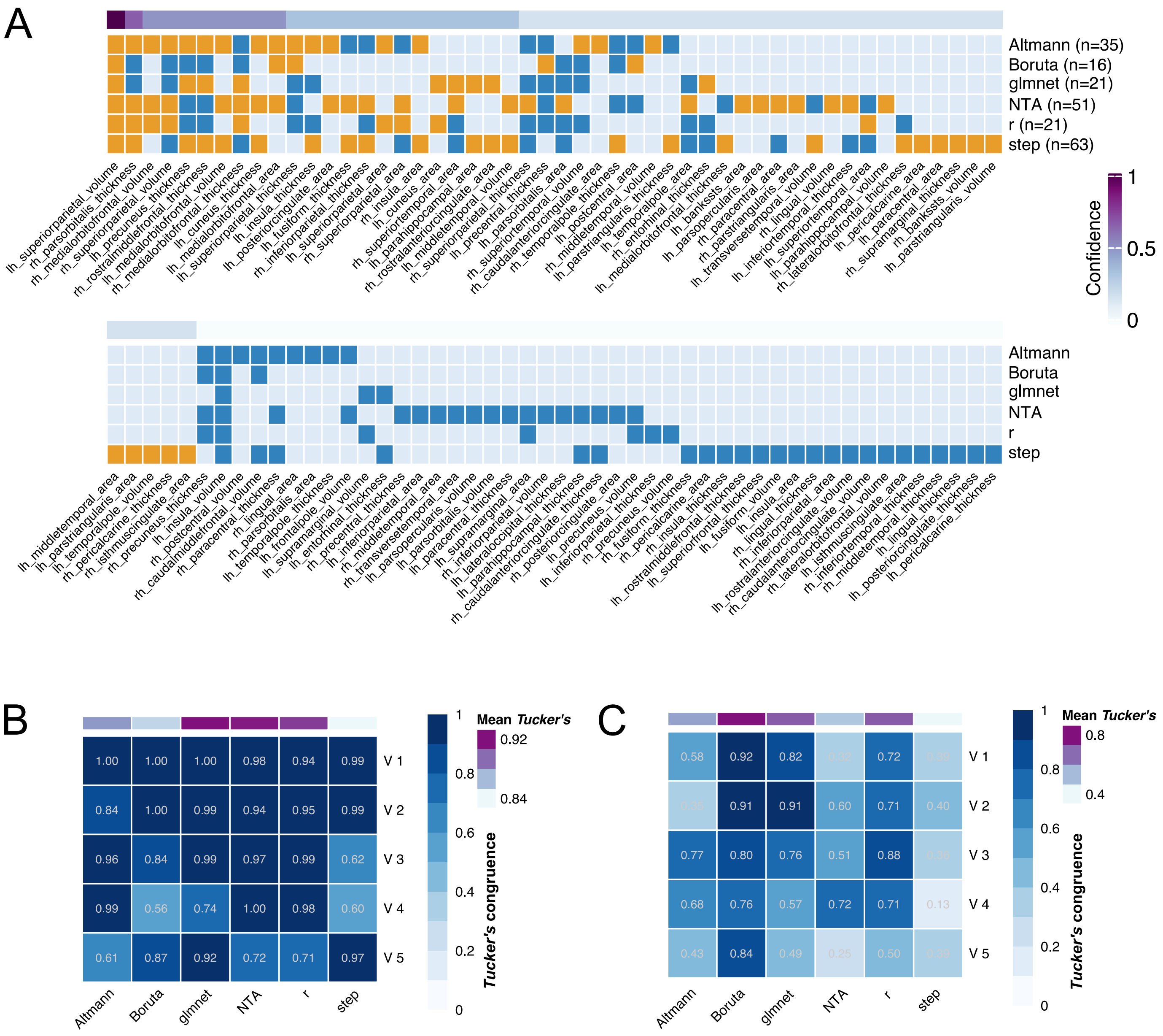

(A) Subsets of neuroanatomical features selected by different feature selection algorithms. A dark blue rectangle indicates that a feature was selected to be of clinical importance but did not display a significant difference in loadings between carriers and non-carriers of the 22q11.2 microdeletion. An orange rectangle indicates that a feature was selected as being clinically relevant AND displayed a significant difference in loading between groups. The blue to purple colorbar indicates the level of confidence associated with the variable selection and between-group difference in loading across selection algorithms. (B) Similarity in the clinical factor between groups based on the subset of neuroanatomical features provided by the different feature selection algorithms. (C) Similarity in the neuroanatomical factor structure across groups for different feature selection algorithms. For (B) and (C), the blue colorbar indicates the *Tucker's congruence coefficient* based on the variable loadings on the different canonical variates (*V*). The purple colormap indicates the mean Tucker's congruence coefficient across canonical variates.

Supplementary Figure S9. *Boxplots for the significant 22q11.2DS-by-ASD Interaction Clusters*

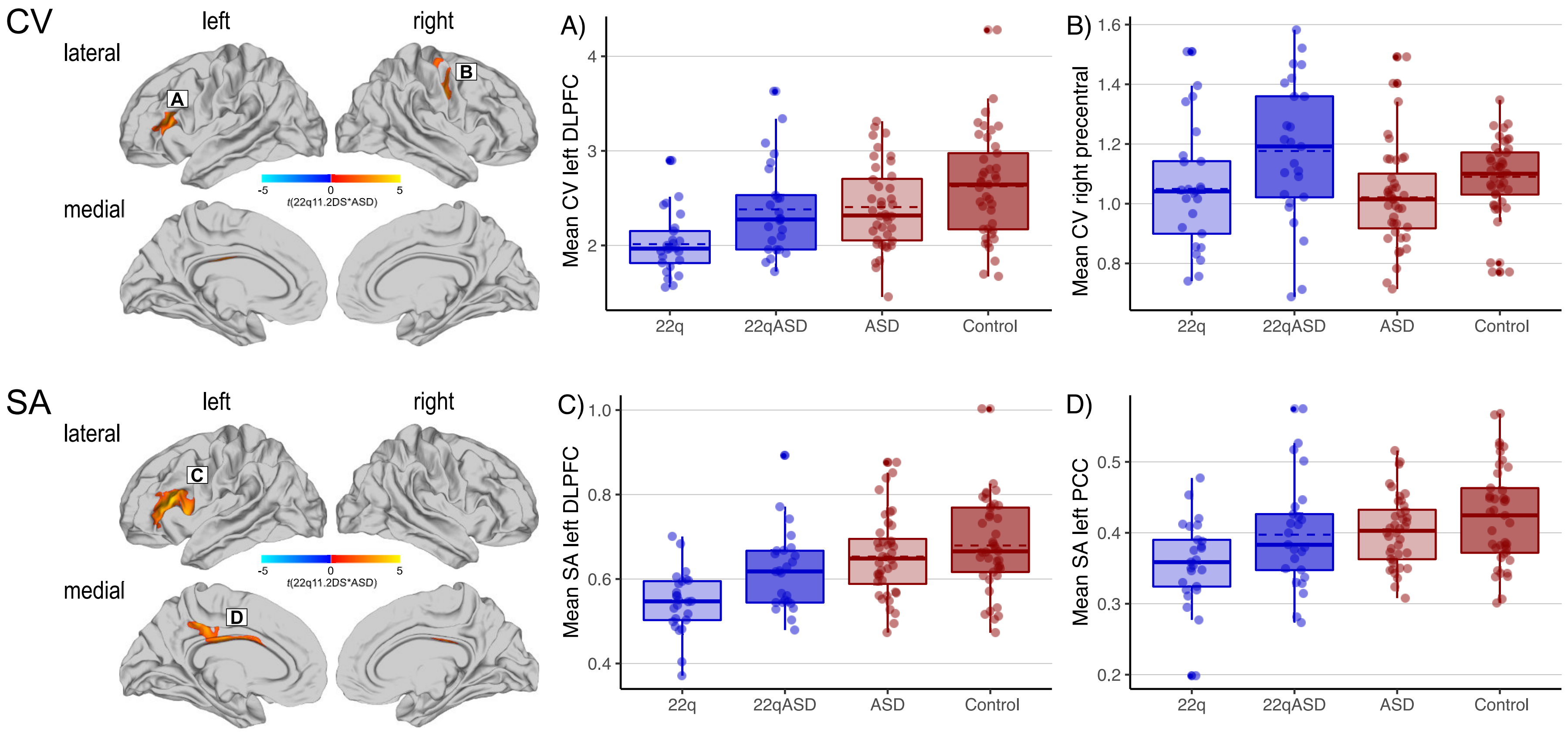

Boxplots displaying the significant 22q11.2DS-by-ASD interaction effects for cortical volume (CV) in the left dorsolateral prefrontal cortex (DLPFC; BA 44-46; A) and the right precentral gyrus (BA 4; B); and for surface area (SA) in the left DLPFC (BA 44-46; C) and the left posterior cingulate cortex (PCC; BA 23-24/31; D). The solid bars indicate the median values of each group, with the lower and upper hinges corresponding to the first (the 25th percentile) and third (the 75th percentile) quartiles, while the dashed bars represent the mean values. '22q' represents 22q11.2 deletion carriers without ASD symptomatology, '22qASD' represents 22q11.2 deletion carriers with ASD symptomatology, 'ASD' represents individuals with idiopathic ASD, and 'Control' represents typically developing controls.

Supplementary Figure S10. *Categorical 22q11.2DS-by-ASD Interaction Effect when covarying for repetitive symptoms*

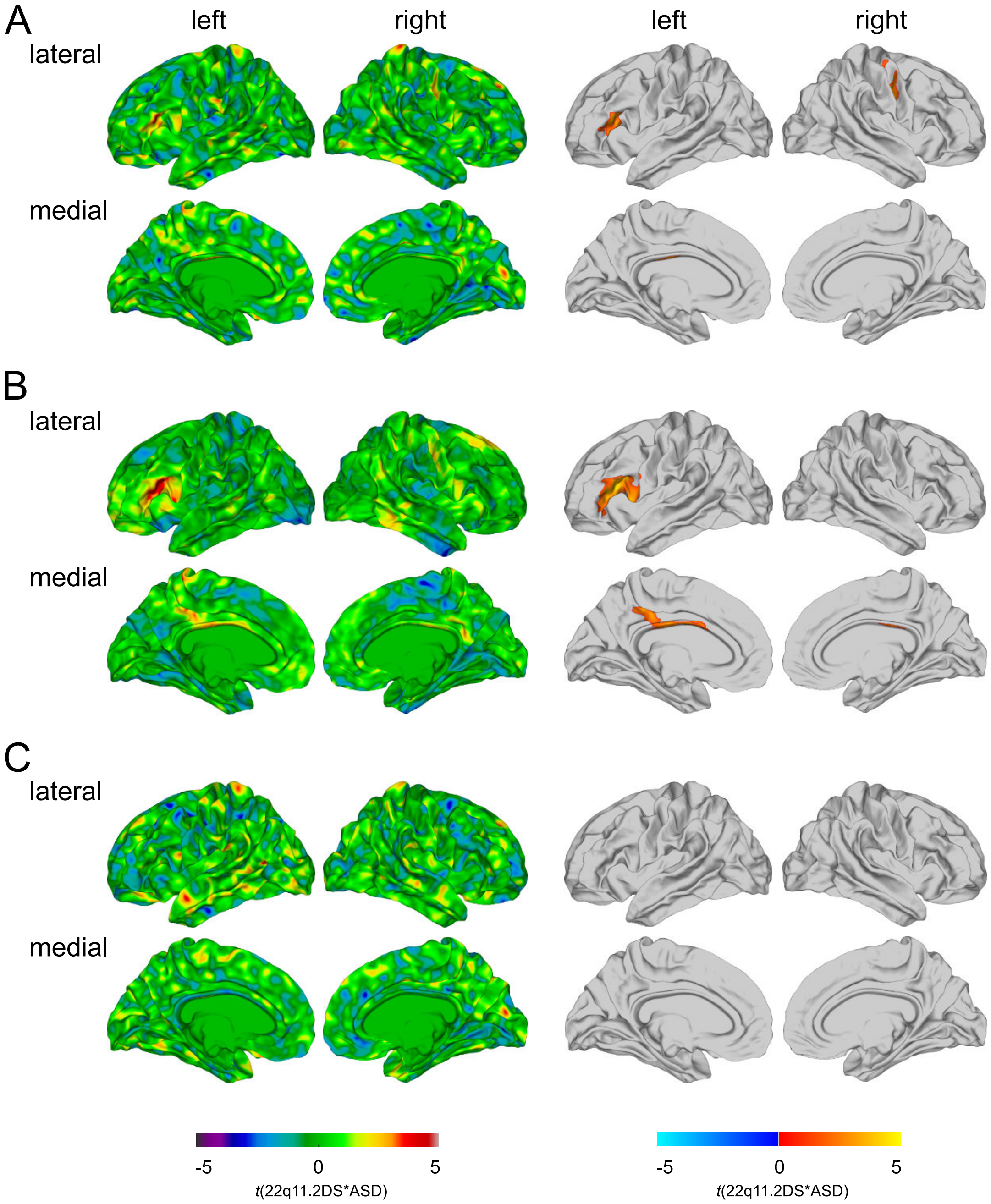

Significant 22q11.2DS-by-ASD interaction effects for cortical volume (CV; A), surface area (SA; B), and cortical thickness (CT; C), when covarying for repetitive symptoms based on the Social Responsiveness Scale (SRS) Restricted Interests and Repetitive Behavior subscale. Displayed are the un-thresholded *t*-maps (left panel) and the random field theory (RFT)-based cluster corrected (p<0.05, 2-tailed) difference maps following multiple comparisons (right panel).

Supplementary Figure S11. *Distribution of SRS subdomain and total scores across groups*

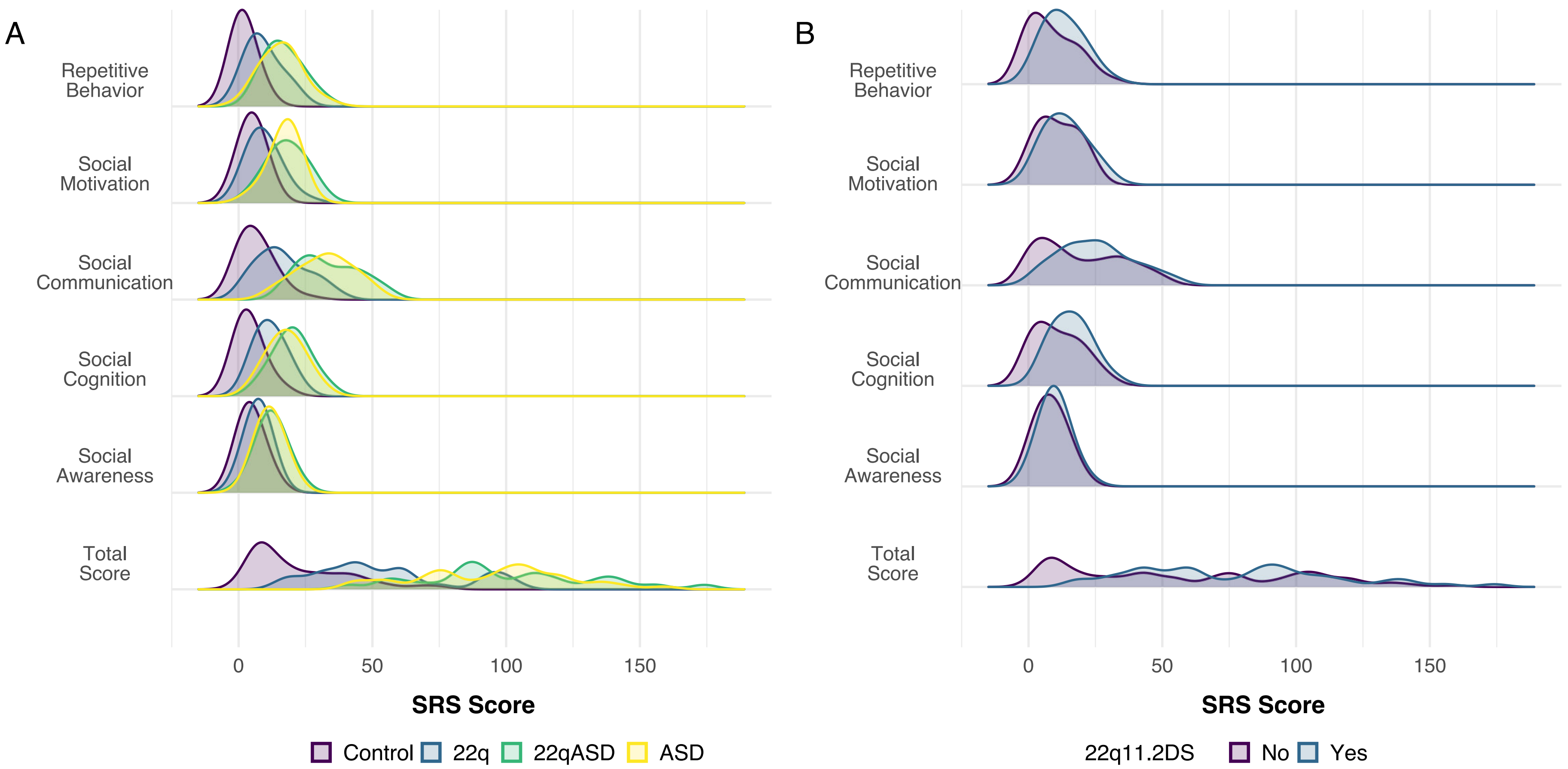

Histograms indicating the distribution of Social Responsiveness Scale scores between groups. Displayed are both the subdomain and total scores between groups (A) for typically developing (TD) controls (Control; purple), individuals with 22q11.2DS without ASD (22q; blue), individuals with 22q11.2DS and ASD (22qASD; green), and idiopathic ASD individuals (ASD; yellow), as well as comparison of (B) individuals with 22q11.2DS (i.e. 22q and 22qASD; purple) compared to those without (i.e. TD controls and ASD; blue).

References

1. Gotham K, Pickles A, Lord C. Standardizing ADOS Scores for a Measure of Severity in Autism Spectrum Disorders. J Autism Dev Disord. 2009;39(5):693-705.

2. Hus V, Lord C. The Autism Diagnostic Observation Schedule, Module 4: Revised Algorithm and Standardized Severity Scores. J Autism Dev Disord. 2014;44(8):1996-2012.

3. Hus V, Gotham K, Lord C. Standardizing ADOS Domain Scores: Separating Severity of Social Affect and Restricted and Repetitive Behaviors. J Autism Dev Disord. 2014;44(10):2400-12.

4. Harrell F. Regression modeling strategies: with applications to linear models, logistic regression, and survival analysis. New York: Springer-Verlag New York; 2013.

5. Kursa MB, Rudnicki WR. Feature Selection with the Boruta Package. 2010. 2010;36(11):13.

6. Friedman J, Hastie T, Tibshirani R. Regularization Paths for Generalized Linear Models via Coordinate Descent. Journal of statistical software. 2010;33(1):1-22.

7. Altmann A, Toloşi L, Sander O, Lengauer T. Permutation importance: a corrected feature importance measure. Bioinformatics. 2010;26(10):1340-7.

8. Janitza S CE, Boulesteix A.L. A computationally fast variable importance test for random forest for high dimensional data. University of Munich; 2015.

9. Haufe S, Meinecke F, Görgen K, Dähne S, Haynes J-D, Blankertz B, et al. On the interpretation of weight vectors of linear models in multivariate neuroimaging. Neuroimage. 2014;87:96-110.

10. Tucker LRC. A method for synthesis of factor analysis studies. Washington, D. C.: Department of the Army, 1951 (Mimeographed); 1951.

11. Lorenzo-Seva U, Berge J. Tucker's Congruence Coefficient as a Meaningful Index of Factor Similarity. Methodology: European Journal of Research Methods for The Behavioral and Social Sciences. 2006;2:57-64.
